# A Novel Color Change Mechanism for Breast Cancer Biomarker Detection: Naphthoquinones as Specific Ligands of Human Arylamine *N*-Acetyltransferase 1

**DOI:** 10.1371/journal.pone.0070600

**Published:** 2013-08-05

**Authors:** Nicola Laurieri, James E. Egleton, Amy Varney, Cyrille C. Thinnes, Camilo E. Quevedo, Peter T. Seden, Sam Thompson, Fernando Rodrigues-Lima, Julien Dairou, Jean-Marie Dupret, Angela J. Russell, Edith Sim

**Affiliations:** 1 Department of Pharmacology, University of Oxford, Oxford, United Kingdom; 2 Department of Chemistry, Chemistry Research Laboratory, University of Oxford, Oxford, United Kingdom; 3 Unit of Functional and Adaptive Biology, University Paris-Diderot, Sorbonne Paris Cité, BFA, EAC 4413 CNRS, 75205 Paris, France; 4 Faculty of Science, Engineering and Computing, Kingston University, Kingston, United Kingdom; Health Canada and University of Ottawa, Canada

## Abstract

Human arylamine *N*-acetyltransferase 1 (hNAT1) has become an attractive potential biomarker for estrogen-receptor-positive breast cancers. We describe here the mechanism of action of a selective non-covalent colorimetric biosensor for the recognition of hNAT1 and its murine homologue, mNat2, over their respective isoenzymes, leading to new opportunities in diagnosis. On interaction with the enzyme, the naphthoquinone probe undergoes an instantaneous and striking visible color change from red to blue. Spectroscopic, chemical, molecular modelling and biochemical studies reported here show that the color change is mediated by selective recognition between the conjugate base of the sulfonamide group within the probe and the conjugate acid of the arginine residue within the active site of both hNAT1 and mNat2. This represents a new mechanism for selective biomarker sensing and may be exploited as a general approach to the specific detection of biomarkers in disease.

## Introduction

Diagnosis of breast cancer combines non-invasive examinations, such as mammography, ultrasound or magnetic resonance imaging and biopsy tests. At the present time, the analysis of biological samples allows the identification of tumor-specific biomarkers to stratify anti-target therapies [1]–[3]. A variety of chemical approaches have been developed to selectively detect and monitor biomolecules, and to generate novel molecular sensors for biological markers to facilitate diagnosis with improved accuracy [4]–[7].

Proteomic and microarray analyses have identified the overexpression of *human arylamine* N*-acetyltransferase 1* (*hNAT1*) in estrogen-receptor-positive ductal and lobular breast cancers [8]–[10] and more recently in male breast cancers [11]; furthermore, this overexpression inversely correlates to tumor grade [12]. In addition to its catalytic role as an arylamine-metabolizing enzyme using acetyl coenzyme A (AcCoA) as cofactor [13] subsequent studies have assessed hNAT1 as a new biomarker to be developed as a novel diagnostic, prognostic and potential therapeutic target in breast cancers [10]–[11].

**Figure 1:**
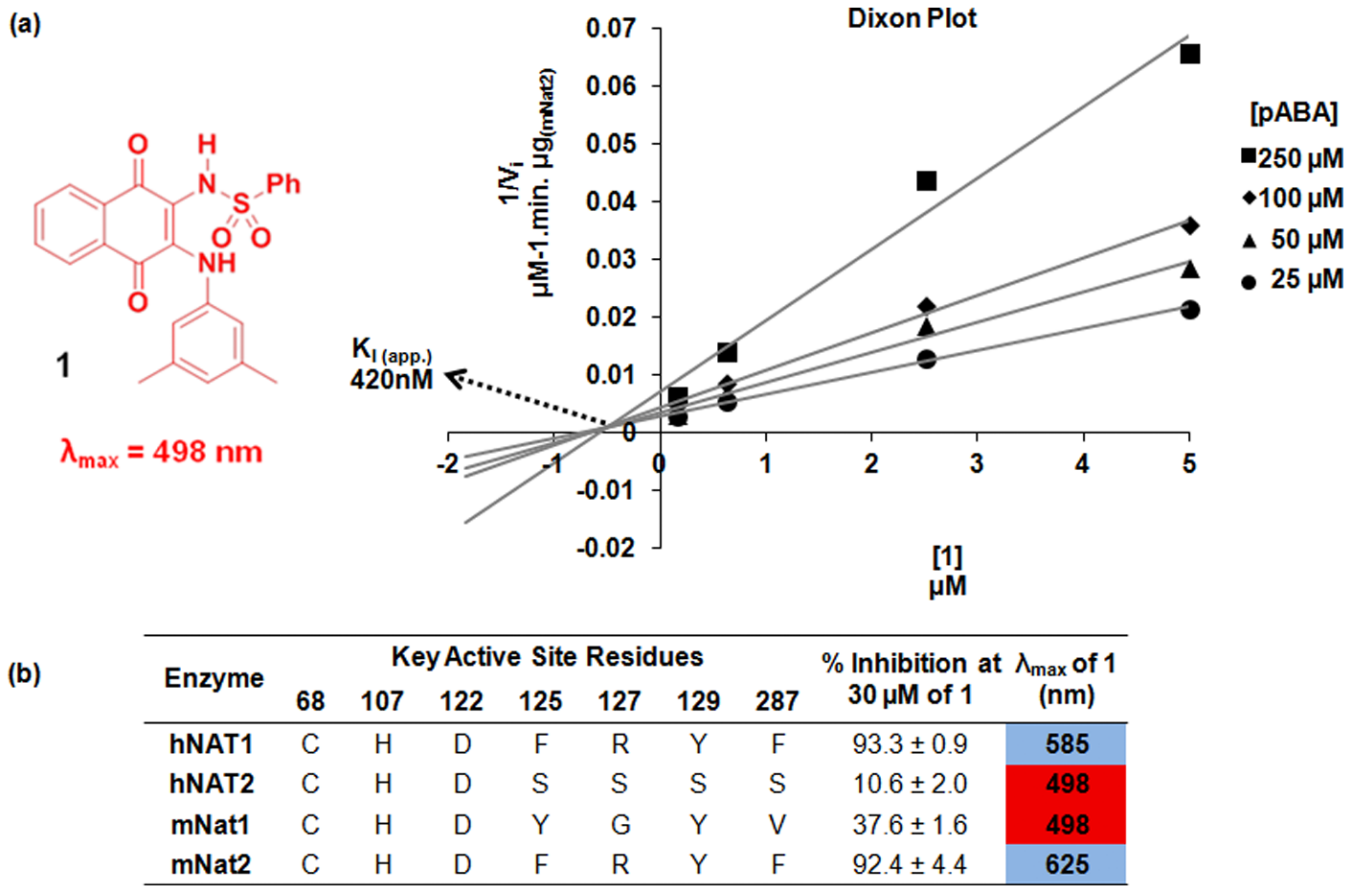
Competitive inhibition of 1 towards mNat2 and active site differences in mammalian NATs (**a**) **Left panel**: Structure of compound **1**; **Right panel**: Dixon plot shows competitive inhibition of mNat2 (9 ng) by **1** at different pABA concentrations (25 µM (circles), 50 µM (triangles), 100 µM (diamonds), and 250 µM (squares)). Initial rates of the mNat2 catalysed reaction were determined by monitoring the rate of hydrolysis of AcCoA (400 μM) (**b**) Summary table of active site differences of human and murine NATs and the effects of their interaction with **1**. Blue and red columns indicate the color of **1** on interaction with the protein.

We have previously described a family of naphthoquinones as non-covalent competitive selective inhibitors of hNAT1 and its murine homologue mNat2 over other eukaryotic and prokaryotic isoforms [14]–[16]. Remarkably, an instantaneous distinctive color change from red to blue is observed upon binding of these naphthoquinone ligands, such as compound **1**, to both hNAT1 and mNat2, which share more than 80% identity in amino acid sequence and are functionally homologous (**Figure 1**) [16]–[17]. No such shift in the λ_max_ of naphthoquinone **1** was observed in the presence of the other human and murine NAT enzymes despite the high number of identical residues (>70%) (**Figure 1**), nor with NATs from prokaryotes [16]. Since hNAT1 is a candidate biomarker in breast cancer, it was reasoned that understanding the mechanism of recognition and color change between this family of naphthoquinone probes and hNAT1 could allow both the development of these probes for tumor subtype diagnosis and the application of this technology to other protein families.

The observed color change was proposed to be caused by selective recognition of the conjugate base of the naphthoquinone mediated by an appropriate residue within the active site of hNAT1 or mNat2, since a similar bathochromic shift in the λ_max_ of naphthoquinone **1** was also observed in an alkaline solution [16]. Kinetic studies with pure recombinant mNat2 have revealed a competitive mode of inhibition of **1** towards the arylamine substrate *para*-amino benzoate (K_i,app._  = 420 nM), which is consistent with previous studies using another member of this naphthoquinone family (**Figure 1** **and Figure S1 in File S1**) [16].

We describe a set of spectroscopic, chemical, molecular modeling and biochemical studies to interrogate the key molecular interactions between hNAT1 or its murine homologue, mNat2, and naphthoquinone **1** which lead to the observed color change event.

## Results and Discussion

Reduction of the naphthoquinone core of **1** has previously been examined and discounted as a possible mechanism for this color change [16]. Additionally, it was found that the visible spectrum of **1** when treated with 1,8-diazabicyclo[5.4.0]undec-7-ene (DBU), a non-nucleophilic amidine base, is comparable to that of **1** in the presence of aqueous NaOH (**Figure S2 in File S1**). This strongly suggests that the observed color change follows an acid-base interaction rather than a potential nucleophilic addition mechanism to the electrophilic enone system within **1**.

Based on the hypothesis that selective recognition of the conjugate base of **1** within the hNAT1 or mNat2 active site is responsible for the observed color change, we aimed to identify both the acidic proton within **1** and the suitably located basic residue within the enzyme active site.

An acid-base titration revealed the pK_a_ of **1** to be ∼9.2 in 5% DMSO/95% H_2_O (**Figure S3 in File S1**), which is consistent with the deprotonation of the sulfonamide moiety of **1**. Although the sulfonamide-N*H* could be identified in the ^1^H-NMR spectrum of **1** in 100% DMSO-*d_6_* solution (δ_H_  = 9.05 ppm) [16], adding as little as 5% D_2_O to the DMSO-*d_6_* solvent promoted rapid proton-deuteron exchange. Direct observation of the sulfonamide-N*H* resonance by ^1^H-NMR spectroscopy could not be carried out under aqueous alkaline conditions nor in the presence of a NAT isoform under the assay conditions required for protein integrity (5% DMSO-*d_6_*/95% D_2_O).

**Figure 2:**
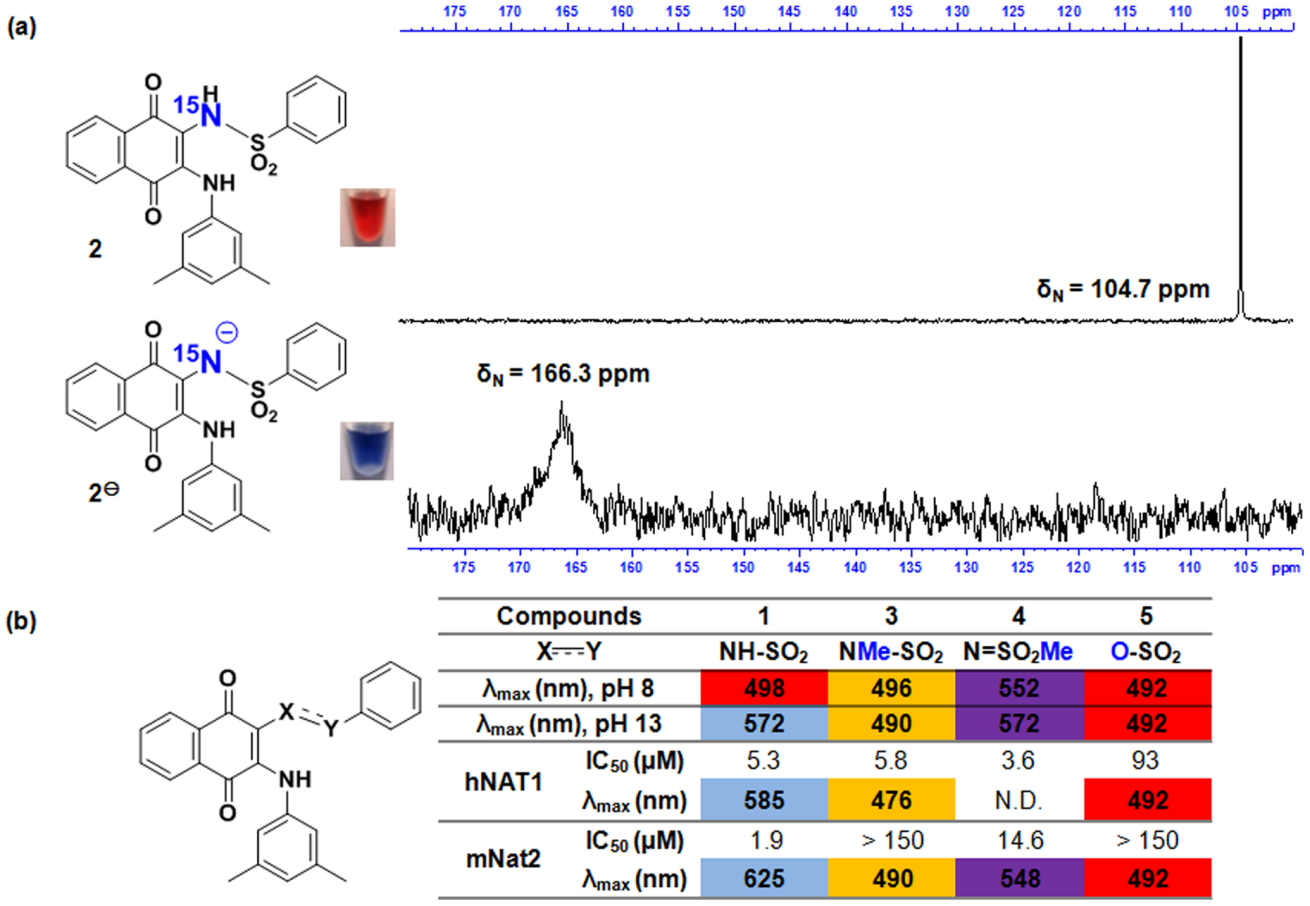
Requirement for the acidic proton of sulfonamide-N*H* for the color change event. (**a**) **Upper:** ^15^ *N-*NMR spectrum of **2** in 95% DMSO-*d_6_*/5% H_2_O. Image of 50 μL of **2** (2 mM) in DMSO with 10 μL of 20 mM Tris.HCl, pH 8. **Lower:** ^15^ *N-*NMR spectrum of **2** in 95% DMSO-*d_6_*/5% aq. NaOD (final NaOD concentration 75.8 mM). Image of 50 μL of **2** (2 mM) in DMSO with 10 μL of 4 mM NaOH, pH 13. (**b**) Comparison of the colorimetric and inhibitory properties of compounds **1**, **3**, **4** and **5** towards hNAT1 and mNat2. Colors of cells indicate the observed color of the relevant compound under the given conditions.

The corresponding ^15^N-labelled sulfonamide analogue of **1** was thus synthesized (compound **2**) to monitor the sulfonamide-*N*H using ^15^N-NMR spectroscopy. The ^15^N-NMR spectrum of **2** under alkaline conditions (95% DMSO-*d_6_*/5% aqueous NaOD) was acquired and compared to the spectrum in 100% DMSO-*d_6_* (**Figure 2a**). The ^15^N spectrum of **2** under neutral conditions displayed a sharp resonance at 104.7 ppm, whilst the spectrum under alkaline conditions showed a broader resonance at 166.3 ppm. The displacement of the ^15^N chemical shift is consistent with the electron density changing around the sulfonamide-^15^N in an alkaline environment. It was not however possible to acquire ^15^N-NMR spectra in the presence of hNAT1 or mNat2 under conditions in which the protein would be active (5% DMSO-*d_6_*/95% D_2_O) as **2** was insufficiently soluble.

Therefore, an alternative approach was adopted, whereby a close analogue of **1** was synthesized incorporating an *N-*methyl-*N*-sulfonyl moiety to determine the effect of removing the possibility of sulfonamide deprotonation with minimal disruption to other structural and chemical properties of the molecule. Treatment of **1** with one equivalent of TMS-diazomethane produced a separable mixture of the *N*-methyl substituted species **3** and the *O*-methyl substituted species **4**, which were isolated in 55% and 35% yields respectively (see **File S1**).

Compound **3** was found to be a good inhibitor of hNAT1 (IC_50_ = 5.8 µM), but a very poor inhibitor of mNat2 (IC_50_ >150 µM), whilst **4** inhibited both enzymes with reasonable potency (IC_50_ = 3.6 µM for hNAT1 and IC_50_ = 14.6 µM for mNat2) (**Figure 2b**). Visible spectra of the *N*-methylated species **3** show that no color change occurs either in aqueous NaOH solution or in the presence of hNAT1 or mNat2. Visible spectra of the *O*-methylated species **4** also do not show an instantaneous shift in λ_max_ in the presence of mNat2 (**Figure 2b** **and Figures S4 and S5 in File S1**). Neither **3** nor **4** was active against the other human NAT isoenzyme, hNAT2, as more than 90% of enzyme activity was retained at an inhibitor concentration of 30 µM.

Sulfonate ester **5** was also synthesized, since this compound was predicted to have similar steric and electronic properties to **1** but lacks an acidic sulfonamide-N*H*. As predicted, **5** does not undergo a color change in the presence of either aqueous NaOH or in the presence of either hNAT1 or mNat2 (**Figure 2b** **and Figure S6 in File S1**). Compound **5** was observed to be a weak inhibitor of hNAT1 (IC_50_ = 93 µM) and a very poor inhibitor of mNat2 (IC_50_ >150 µM).

With structure-activity-relationship data in hand supporting the key role of sulfonamide deprotonation in the color change event, structural-based site-directed mutagenesis studies were next undertaken to establish which amino acid residue within the active site of hNAT1 or mNat2 is able to recognize the conjugate base of the sulfonamide moiety within **1**.

**Figure 3:**
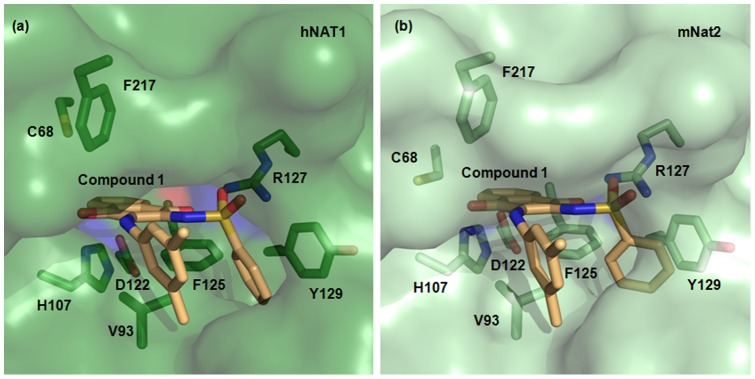
Inhibitor binding pocket of hNAT1 and mNat2. (**a**) The active site of hNAT1 crystal structure (PDB:2PQT) in surface representation with **1** docked in stick representation. The hNAT1 residues involved in inhibitor binding and selectivity are shown in stick representation and labeled with carbon in green, nitrogen in blue, oxygen in red, and sulfur in yellow. **1** is labeled with carbon atoms in light orange, nitrogen in blue, oxygen in red, and sulfur in yellow. (**b**) The active site of mNat2 structural model with docked compound **1** is shown using the same representation as in (a).

X-Ray crystal structures of both human NAT enzymes are available [18]; hNAT1 (PDB:2PQT) was used to build a structural model for mNat2. **1** was computationally docked within the active site of hNAT1 (**Figure 3a**) and into the mNat2 structural model (**Figure 3b**). The results from both docking studies were compared and several distinctive interactions between **1** and hNAT1 or mNat2 were predicted.

The close proximity of the sulfonamide functionality of the ligand and the Arg127 guanidine of both enzymes in the docking models suggested this residue was important for mutual recognition. In particular, both a sulfonamide oxygen atom and the carbonyl group at C_1_ of the naphthoquinone core of **1** appear to be able to form a hydrogen bond with the Arg127 guanidine group in the range of 2.5–3.0 Å. Meanwhile, the other sulfonamide oxygen appears close in space to the backbone amide carbonyl of Phe287 (2.5 Å).

Further notable potential interactions include the hydrophobic van der Waals interactions between the naphthoquinone core of **1** and the hydrophobic plane defined by the side chain arene of Phe125 and the isopropyl side chain of Val93 (3.5–4.0 Å); and additionally, the parallel-displaced π-π stacking between the C_2_ sulfonamide arene of **1** and the side chain arene of Tyr129 (4.2–4.8 Å).

The unique presence of the triad Phe125, Arg127 and Tyr129 in both hNAT1 and mNat2 compared to other eukaryotic and prokaryotic isoenzymes strongly supports the key role of these residues in selective ligand binding. This observation is consistent with previous findings on the crucial role played by the same residues on arylamine substrate preference [18]–[20]. Arg127 is not found in any of the other NAT enzymes investigated here (**Figure S7 in File S1**).

It was important to rule out the involvement of the catalytic Cys68 residue in mediating the color change event. Selective alkylation of the Cys68 thiolate within the mNat2 active site by incubating mNat2 with iodoacetamide inactivates the enzyme, but does not prevent the ligand 1 from changing color in the presence of the modified enzyme. This provides evidence to suggest that Cys68 is not the residue responsible for the color change (**Figures S8 and S9 in File S1**).

Arg127, which is likely to be protonated under the assay conditions (pH 8), is therefore anticipated to interact with the conjugate base of the sulfonamide moiety on binding of ligand 1, thereby driving the observed color change of 1 from red to blue, as the conjugate base of 1 is sequestered by the enzyme. From a chemical perspective, this accords with the pK_aH_ of a free arginine guanidine (∼12.5) being higher than the pK_a_ of 1 (∼9.2). The proposed mode of recognition between the protein and the ligand generates a strong bidentate ionic interaction between the two counterparts, an important feature contributing to selective recognition and stability of the protein/ligand complex.

Arg127 in mNat2 was therefore mutated by site-directed mutagenesis. Two different mutant *mNat2* constructs were generated after single nucleotide mutation: the first encoding for a Gly at position 127 (*mNat2*_R127G), and the second for a Leu (*mNat2*_R127L, **Figure S10 in File S1**). By substituting Leu in place of Arg, a basic, charged group is removed with only a minor reduction in the predicted active site volume. Substitution for Gly significantly alters the overall size and shape of the active site. Moreover, Gly is the residue present at the same position in the other murine isoenzyme, mNat1. Wild type mNat2 (mNat2_WT) and the two mutants were successfully produced as recombinant proteins and purified with good yields (≥40 mg/L culture) consistent with previous studies on mNat2_WT (**Figures S11 and S12 and** **Table S1 in File S1**) [17].

**Figure 4:**
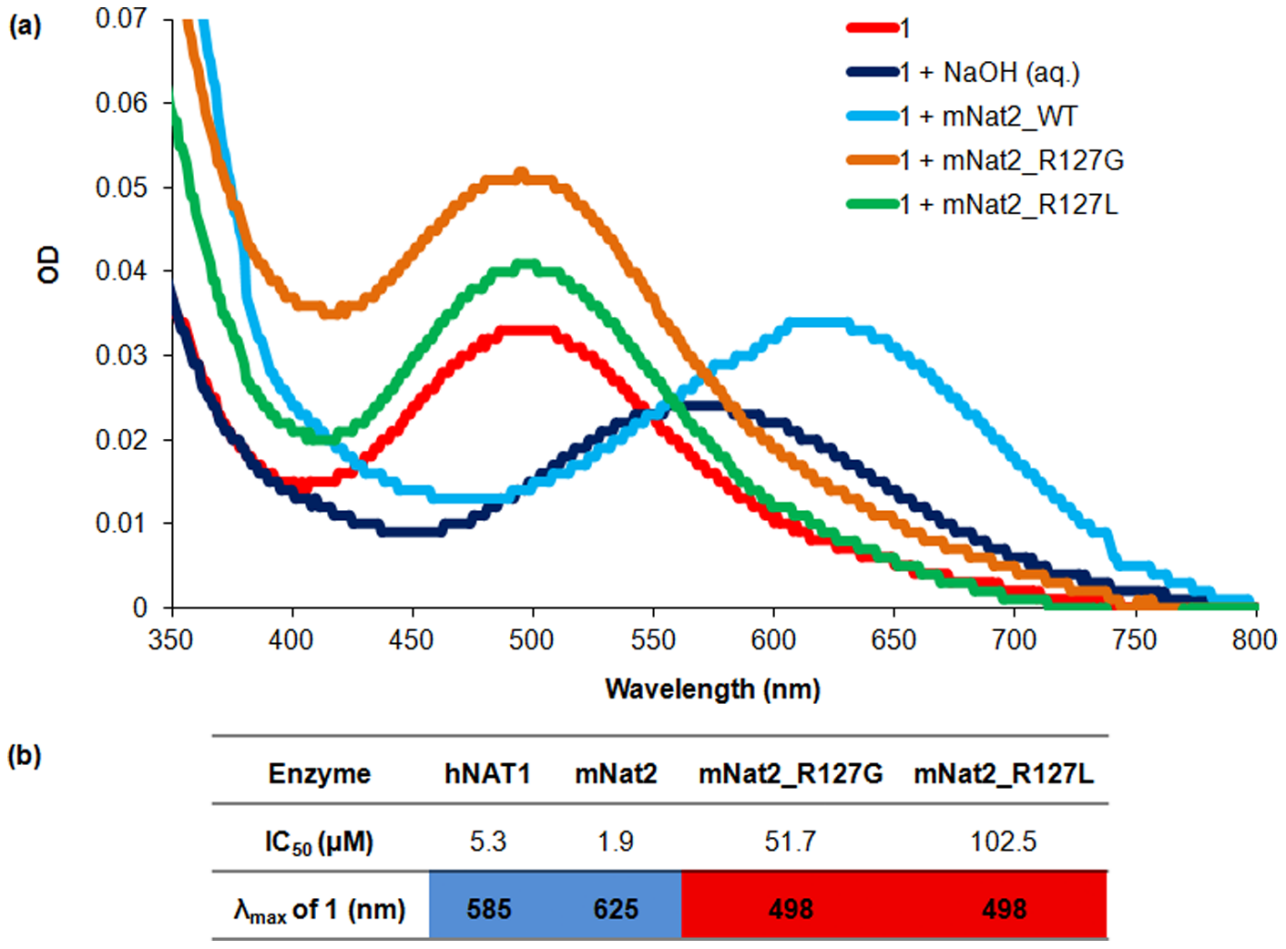
The effects of mutating Arg127 of mNat2 in the interaction with 1. (**a**) Visible spectra of compound **1** (15 μM) incubated under different conditions: 20 mM Tris.HCl at pH 8.0 (red line), with mNat2 variants (30 μM) in 20 mM Tris.HCl at pH 8.0 (mNat2_WT (light blue line), mNat2_R127G (orange line), mNat2_R127L (green line)) or of compound **1** in 80 mM NaOH at pH 13.75 (dark blue line). (**b**) Summary of interactions of **1** with hNAT1, mNat2 and the two engineered mNat2 mutants.

Both site-directed mutants were found to be catalytically active: whilst the R127G mutant has a similar specific activity to the native protein with pABA as substrate (920 nmol/min/mg compared to 850 nmol/min/mg for mNat2_WT), the R127L mutant is less active (13 nmol/min/mg). Each of the mutants are also active against anisidine and showed no activity towards procainamide, like mNat2_WT, suggesting the proteins are appropriately folded to be catalytically active. AcCoA-hydrolysis assays were therefore carried out to investigate their kinetic properties towards naphthoquinone **1**. The activity of each mNat2 mutant enzyme was tested with different concentrations of the inhibitor and IC_50_ curves were obtained and compared (**Figure 4**). The IC_50_ value of **1** with mNat2_WT was 1.9 µM, whereas the IC_50_ values with mNat2_R127G and mNat2_R127L were higher, at 51.7 and 102.5 µM respectively.

Full visible wavelength scans were obtained with **1** in the presence of the three mNat2 variants at pH 8.0. A bathochromic shift was observed with mNat2_WT, but no color change was observed with either mNat2_R127G or mNat2_R127L (**Figure 4**). This supports the hypothesis that Arg127 is key in binding of the ligand to the enzyme and is required for the concomitant color change.

## Conclusions

In summary, the combination of inhibitor structural studies, docking results and mutagenesis studies conducted indicate the color change observed is driven by selective recognition between the conjugate base of the sulfonamide-N*H* of the ligand and the Arg127 guanidininum of the enzyme. The high selectivity and striking colorimetric properties of the naphthoquinone probe **1** towards hNAT1 strongly support the further development of this family of naphthoquinones as selective inhibitors and colorimetric biosensors to target native overexpressed hNAT1 in breast tumors.

## Materials and Methods

### Chemicals and reagents

All chemicals were purchased from Sigma-Aldrich UK, TCI UK, Apollo Scientific UK, Alfa Aesar UK, Fluorochem UK or Fisher Scientific UK unless otherwise stated. Molecular biology reagents were obtained from Promega (Southampton, UK). Competent *E. coli* cells were purchased from Promega and Invitrogen (Carlsbad, USA). The pH of buffer solutions was adjusted at the appropriate temperature.

### Chemical synthesis

The description of the methods for the chemical synthesis of compounds and their characterization data are detailed in the File S1.

### Site-directed mutagenesis and transformation

The pET28b(+) plasmid vector containing the sequence of *mNat2* [17] was isolated from 5 mL overnight bacterial cultures using a QIAprep spin miniprep kit (Qiagen). Site-directed mutagenesis was achieved using QuikChange II kit (Stratagene) to mutate one of the three nucleotides (CGT) encoding for Arg127 in *mNat2*. The reaction mixture (50 μL), which contained 5 μL 10× reaction buffer, 50 ng pET28b(+) plasmid as template, 125 ng of both R127G forward (5′-GCTGGGTTTGGA**GGT**TCCTACCAGATGTGGGAGCC-3′) and R127G reverse (5′-GGCTCCCACATCTGGTAGGA**ACC**TCCAAACCCAGC-3′) primers, or 125 ng of both R127L forward (5′-GCTGGGTTTGGA**CTT**TCCTACCAGATGTGGGAGCC-3′) and R127L reverse (5′-GGCTCCCACATCTGGTAGGA**AAG**TCCAAACCCAGC-3′) primers, and 1 μL dNTP mix, was subjected to thermo-cycling: one cycle of 2 min. at 95°C, 30 cycles of 1 min. at 95°C, 30 s at 60°C, and 1 min. at 72°C, an extra cycle at 72°C for 10 min., and a final cycle at 4°C for 5 min. The un-mutated parental DNA template present in the PCR product was digested by 10 U of *Dpn* I (New England Biolabs) for 1 h at 37°C. Mutant plasmids were verified by sequencing analysis (GeneService at Department of Biochemistry, University of Oxford, UK) to ensure the correct change before transformation by the heat shock method [21] into *E. coli* JM109 and *E.coli* Rosetta(DE3)pLysS strains, as previously described [17].

### Protein production, purification and characterization

All recombinant mouse enzymes including the site-directed mutants of *mNat2* were expressed with a hexa-histidine tag from *E.coli* Rosetta(DE3)pLysS strain transformed with the appropriate plasmid and the protein was then purified *via* Ni-NTA affinity chromatography (Qiagen) and thrombin cleavage of the His-tag, as previously described [17]. Details of the purification steps are shown in SDS page gels and in purification tables reported in the (**Figures S11 and S12 and Table S1 in File S1**). Pure recombinant hNAT1 was produced as previously described [22].

### Enzymatic assays

NAT activity was determined as previously described [15],[23]; a full procedure is outlined in the (**File S1**).

### Colorimetric properties of inhibitors

Visible spectra of each compound were recorded with a U-2001 spectrophotometer (Hitachi) using 1 mL plastic cells of 1 cm path-length (FisherBrand) or 50 μL UVettes® (Eppendorf). Concentrations of inhibitors and protein used are given in the appropriate figure legends. All spectra were blank-corrected.

### Covalent modification of pure recombinant mNat2

To an aliquot of mNat2 (100 µL at 4 mg/mL in 20 mM Tris.HCl, pH 8) was added 5 µL 2-iodoacetamide (0.105 M solution in DMSO) to a final concentration of 5 mM 2-iodoacetamide. The aliquot was incubated at 4°C for 3 h, as previously described [18],[24]–[25]. Enzymatic activity was abolished and MS (MALDI) support covalent modification of Cys68 within mNat2, consistent with previous studies (**Figures S8 and S9 in File S1**, *m/z* 33988 (unmodified enzyme); 34046 (modified enzyme)) [18],[24]–[25].

### Modelling structures

All images showing protein structures were generated using the software PyMOL (W. L. DeLano (2002) PyMOL, DeLano Scientific, San Carlos, CA). A structural model of mNat2 was generated based on the hNAT1 structure (PDB:2PQT) using the on-line software SwissModel, Automated mode (http://swissmodel.expasy.org/) [26]–[28], after removing the acetanilide molecule and restoring the thiol functionality of Cys68. The docking studies on **1** within hNAT1 and mNat2 active sites were conducted as follows. The ligand was first drawn as a 3D structure using the software ChemBio3D Ultra 12.0. The molecular editor Avogadro was used to predict the ground state conformation of the ligand. The analysis of the possible interactions between the protein and the ligand was performed using the licensed software GOLD [29]. A docking site was defined as a region of 10 Å within the active pocket of the enzyme and the ligand was then loaded into the software. The software gave different docked conformers and ranked the generated solutions using the GOLD Score Fitness function [29].

## Supporting Information

File S1 **Synthetic procedures, analytical data, NMR spectra and HPLC traces for all reported compounds, supplementary figures and tables (Figures S1–S12; Table S1) can all be found in File S1.**
(DOCX)
