# Supplementary material for: A Novel Color Change Mechanism for Breast Cancer Biomarker Detection: Naphthoquinones as Specific Ligands of Human Arylamine N-Acetyltransferase 1
Source: PLoS One. 2013 Aug 5;8(8):e70600. doi: 10.1371/journal.pone.0070600 (PMC3734253; doi:10.1371/journal.pone.0070600)
Supplement: File S1 — Synthetic procedures, analytical data, NMR spectra and HPLC traces for all reported compounds, supplementary figures and tables (Figures S1–S12; Table S1) can all be found in File S1. (DOCX) [file pone.0070600.s001.docx]

**A Novel Color Change Mechanism for Breast Cancer Biomarker Detection: Naphthoquinones as Specific Ligands of Human Arylamine *N*-Acetyltransferase 1**

Nicola Laurieri,^a,b,♯^ James E. Egleton,^a,b,♯^ Amy Varney,^a^ Cyrille C. Thinnes,^a,b^ Camilo E. Quevedo,^b^ Peter T. Seden,^b^ Sam Thompson,^b^ Fernando Rodrigues-Lima,^c^ Julien Dairou,^c^ Jean-Marie Dupret,^c^ Angela J. Russell^a,b,^* and Edith Sim^a,d,^*

^a^Department of Pharmacology, University of Oxford, Mansfield Road, Oxford, OX1 3QT, UK

^b^Department of Chemistry, Chemistry Research Laboratory, University of Oxford, Mansfield Road, Oxford, OX1 3TA, UK

^c^Unit of Functional and Adaptive Biology, University Paris-Diderot, Sorbonne Paris Cité, BFA, EAC 4413 CNRS, 75205 Paris, France

^d^Faculty of Science, Engineering and Computing, Kingston University, KT1 2EE, UK

**Supporting Information**

**Supplementary figures**


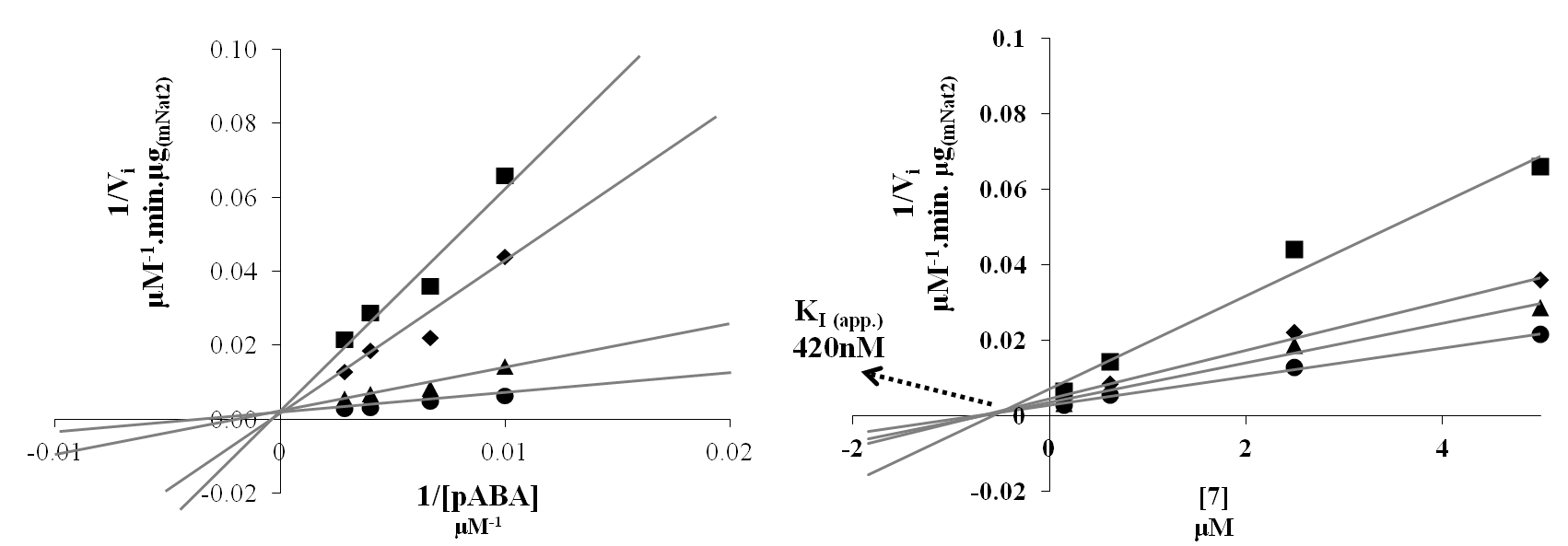


**Figure S1: Kinetic analysis of mNat2 inhibition by compound 1.**

Lineweaver-Burke plot shows activity rates at inhibitor concentrations of 5µM (squares), 2.5µM (diamonds), 0.625µM (triangles) and 0.15625µM (circles). mNat2 initial rates were determined by AcCoA hydrolysis assay using 9 ng of pure recombinant mNat2.


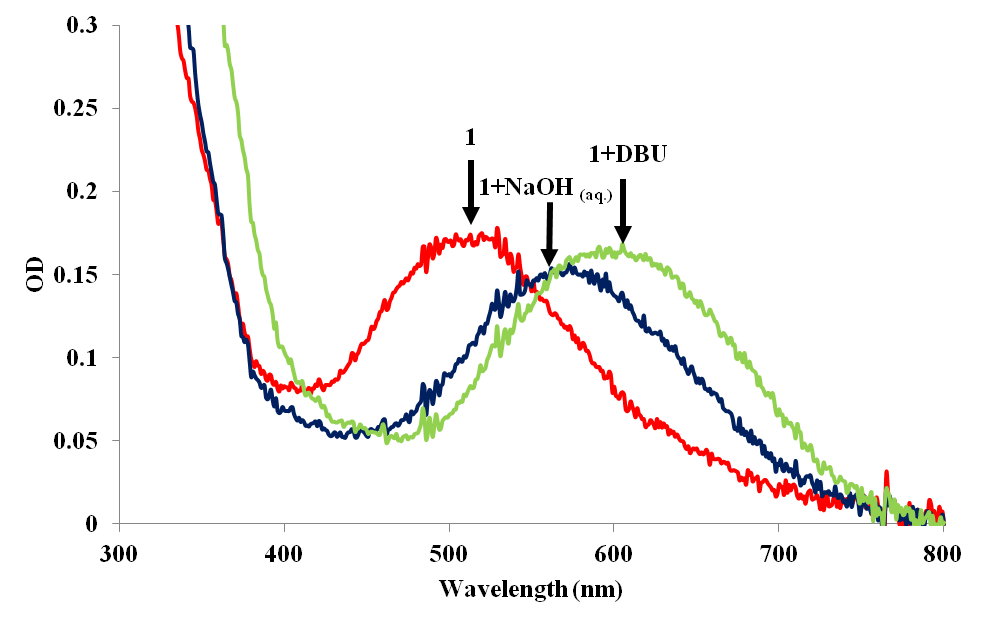


Figure S2: Interaction between 1 and DBU, a non-nucleophilic base.

Visible spectra of 1 (10 μM) in buffer (20 mM aq Tris.HCl, pH 8 containing 5% DMSO (v/v)) (red line), 1 (10 μM) in alkaline solution (80mM aq NaOH containing 5% DMSO (v/v)) (blue line), 1 (10 μM) with DBU (80 mM DBU in DMSO) (green line). All values of optical density were measured against blank buffer solution (20 mM aq Tris.HCl, pH 8 containing 5% DMSO (v/v)).


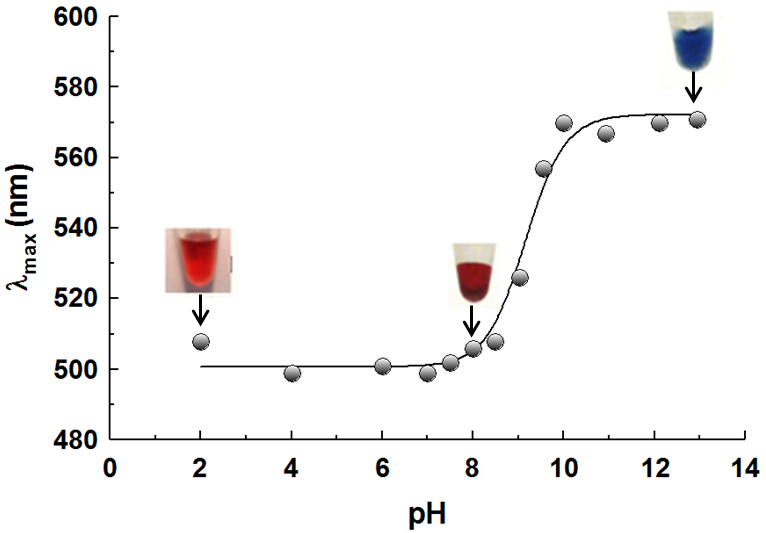


Figure S3: pH titration curve of compound 1.

5 μL of 1 in DMSO (final concentration 0.1 mM) was added to 95 μL of a panel of solutions buffered at diverse pHs in a flat-bottomed 96-well plate (Corning). Visible spectra of the samples were recorded from 800 to 350 nm on the FLUOstar Omega plate reader (BMG Labtech), then the λ_max_ were determined for all spectra and plotted against pH. A sigmoidal titration curve was fitted using GraphPad^®^.


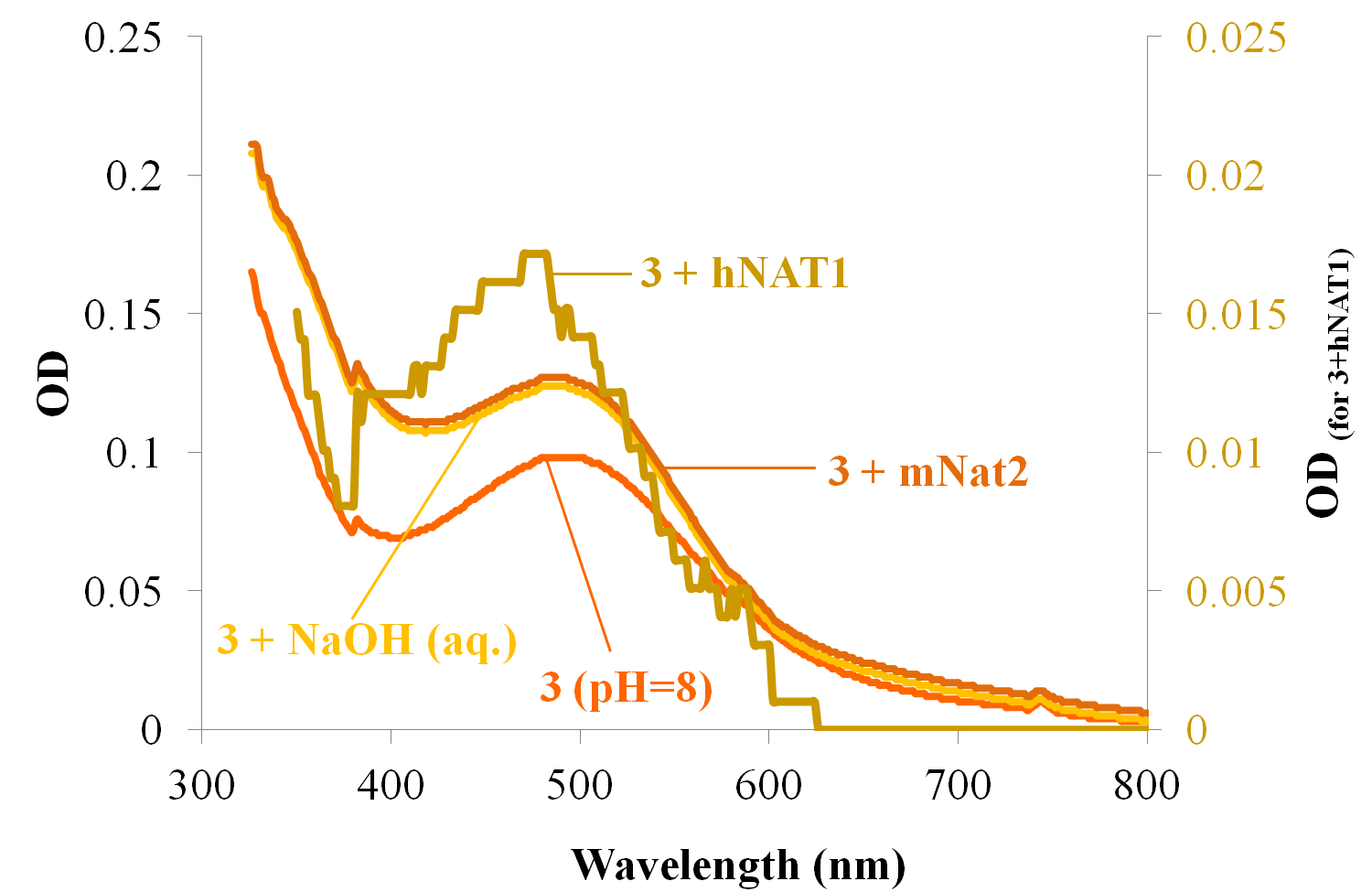


**Figure S4: Colorimetric properties of compound 3.**

15 μM compound **3** was prepared at different conditions: 20 mM aq Tris.HCl, pH 8, 5% DMSO (v/v); 80 mM aq NaOH, pH 13.75, 5% DMSO (v/v); with hNAT1 (30 μM) in 20 mM aq Tris.HCl, pH 8, 5% DMSO (v/v); with mNat2 (30 μM) 20 mM aq Tris.HCl, pH 8, 5% DMSO (v/v). Wavelength scans from 350 to 800 nm were recorded against the respective blanks containing no compound **3**.

**
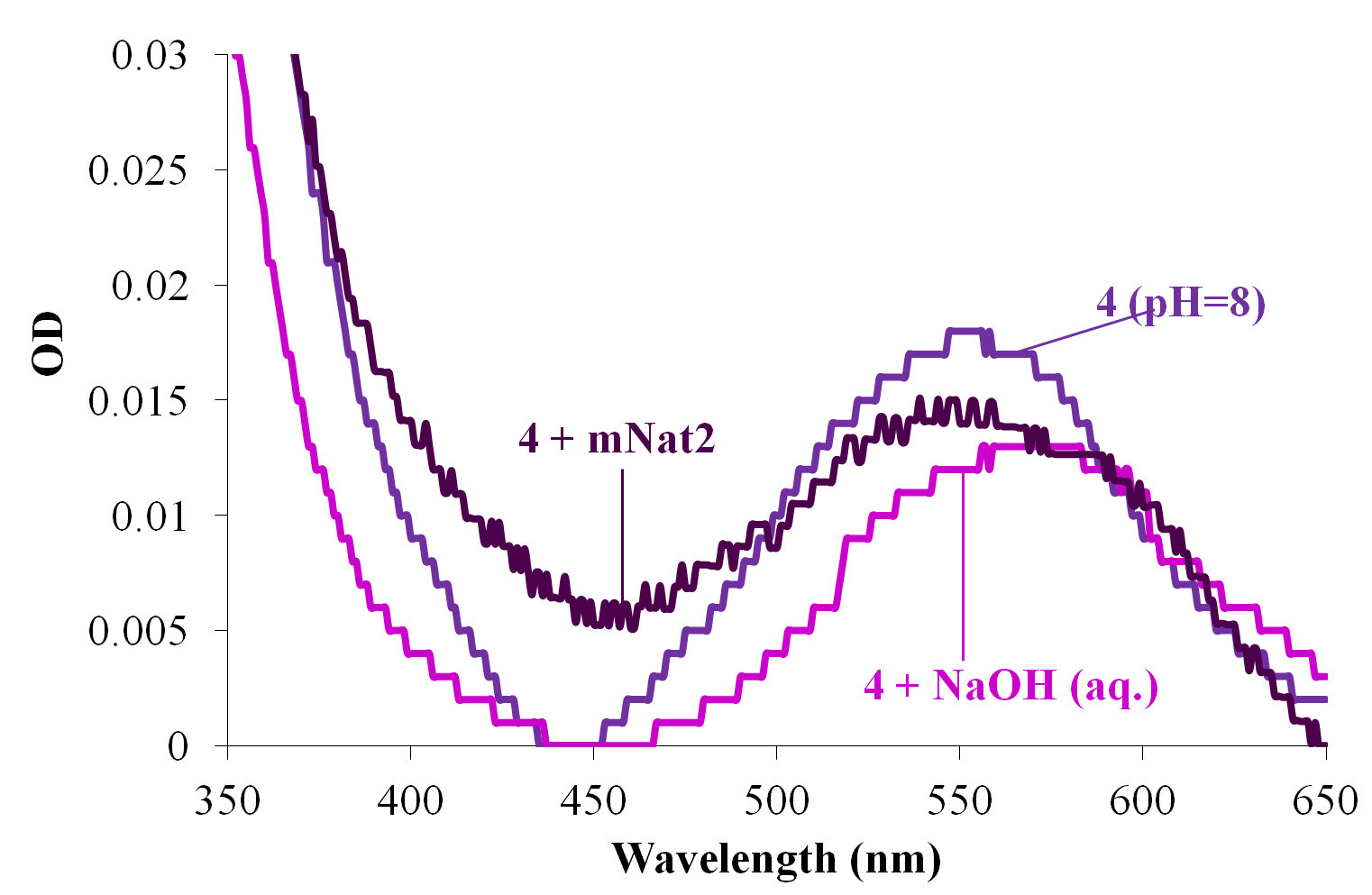
**

**Figure S5: Colorimetric properties of compound 4.**

15 μM compound **4** was prepared using different conditions: 20 mM aq Tris.HCl, pH 8, 5% DMSO (v/v); 80 mM aq NaOH, pH 13.75, 5% DMSO (v/v); with hNAT1 (30 μM) in 20 mM aq Tris.HCl, pH 8, 5% DMSO (v/v); with mNat2 (30 μM) 20 mM aq Tris.HCl, pH 8, 5% DMSO (v/v). Wavelength scans from 800 to 350 nm were recorded against the respective blanks containing no compound **4**.

**
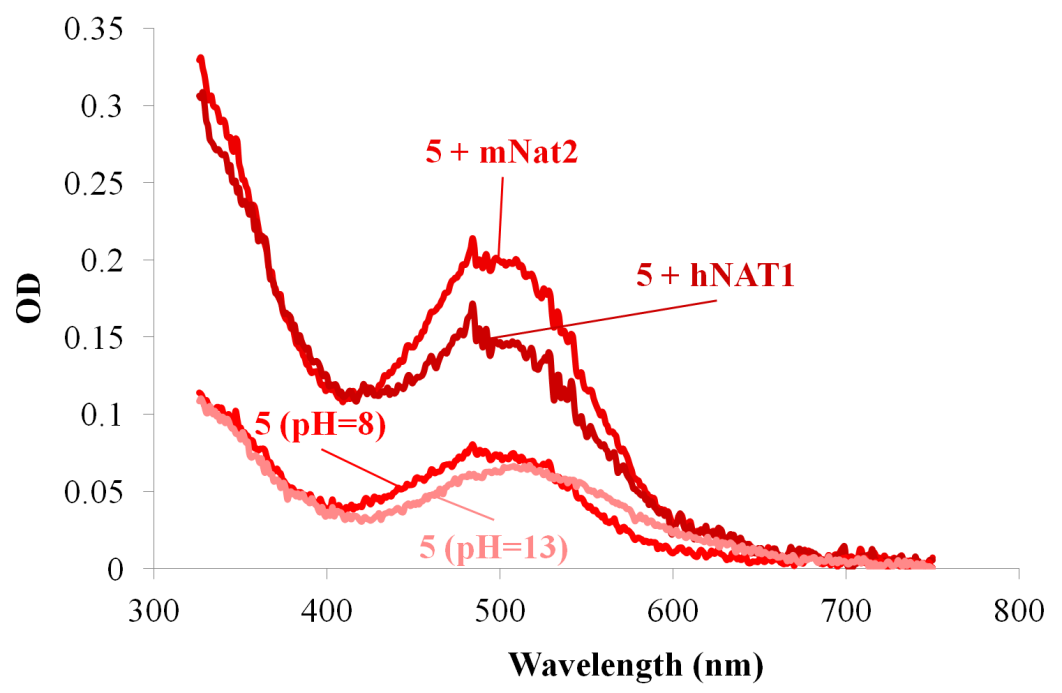
**

**Figure S6: Colorimetric properties of compound 5.**

15 μM compound **5** was prepared using different conditions: 20 mM aq Tris.HCl, pH 8, 5% DMSO (v/v); 80 mM aq NaOH, pH 13.75, 5% DMSO (v/v); with hNAT1 (30 μM) in 20 mM aq Tris.HCl, pH 8, 5% DMSO (v/v); with mNat2 (30 μM) 20 mM aq Tris.HCl, pH 8, 5% DMSO (v/v). Wavelength scans from 800 to 350 nm were recorded against the respective blanks containing no compound **5**.


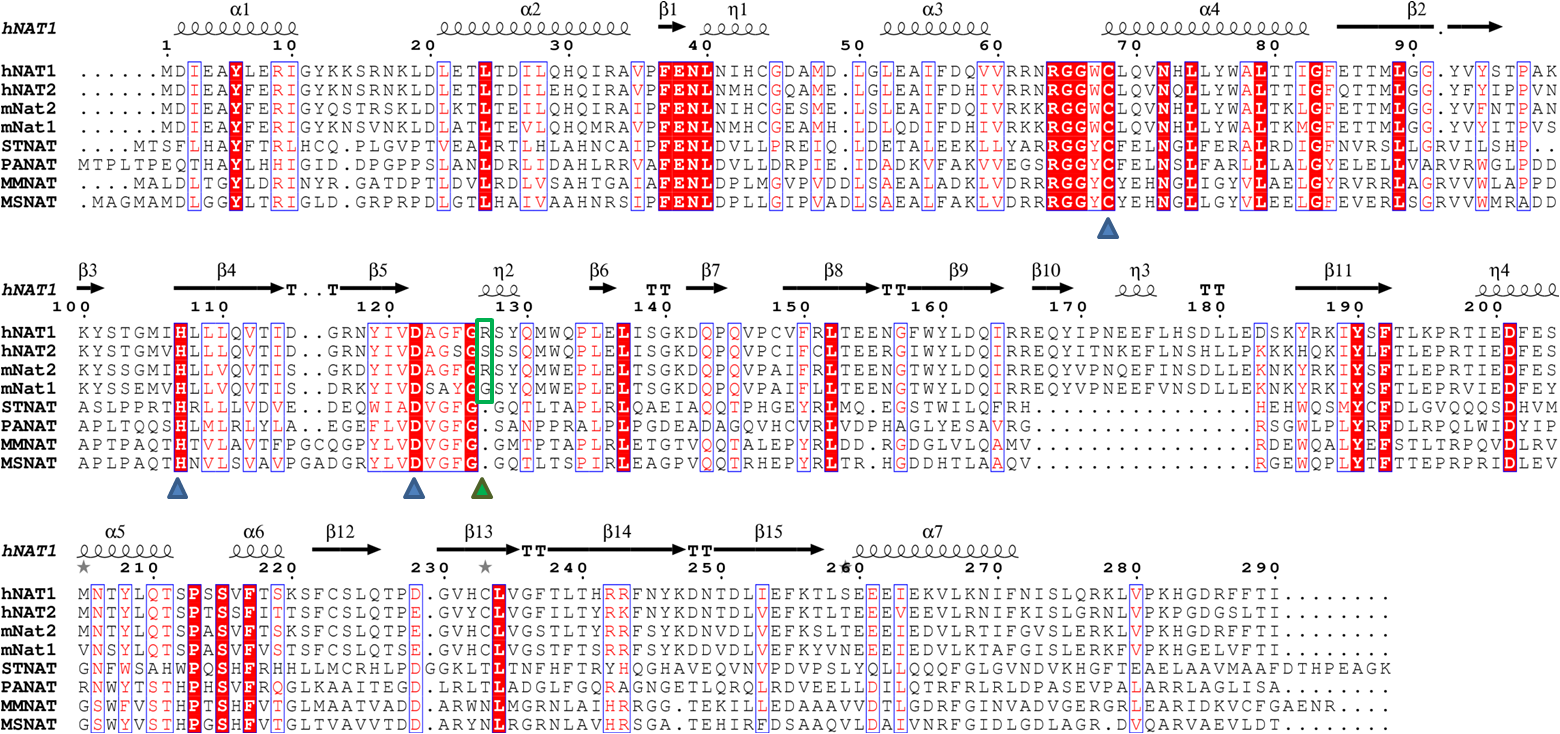


Figure S7: Multiple sequence alignment of selected NAT proteins.

NAT proteins from *S. typhimurium*, *P. aeruginosa*, *M. marinum* and *M. smegmatis* are aligned with murine and human NAT isoenzymes. Similar amino acids are highlighted in blue boxes; completely conserved residues are indicated by white lettering on a red background. The residues from the catalytic triad are indicated by a blue triangle and the putative residue involved in inhibitor selectivity is indicated by a green triangle. The secondary structure is adapted from hNAT1 crystal structure (PDB:2PQT) [1]. Alignment was generated using Clustal W [2]. Figure was prepared using ESPript 2.2 [3].


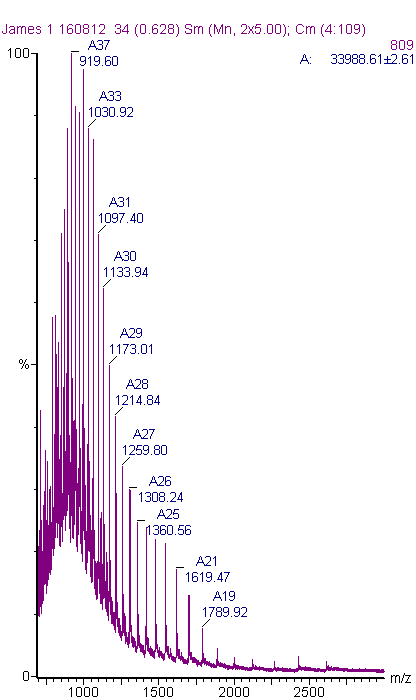

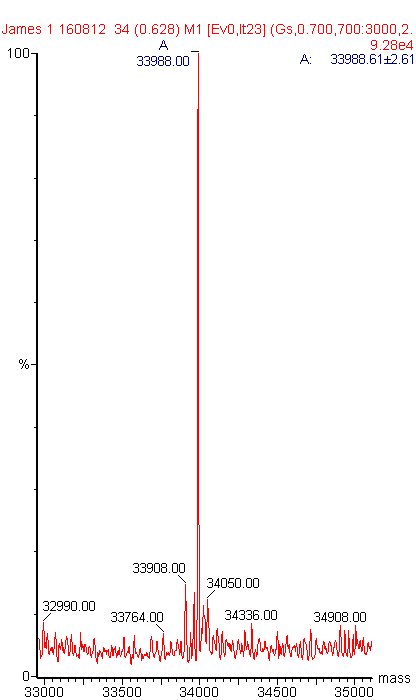


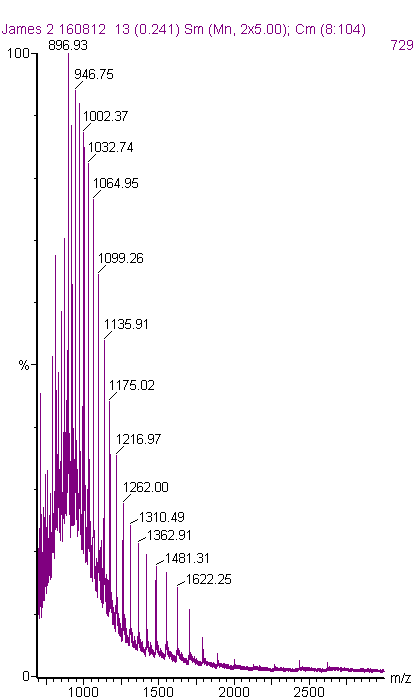

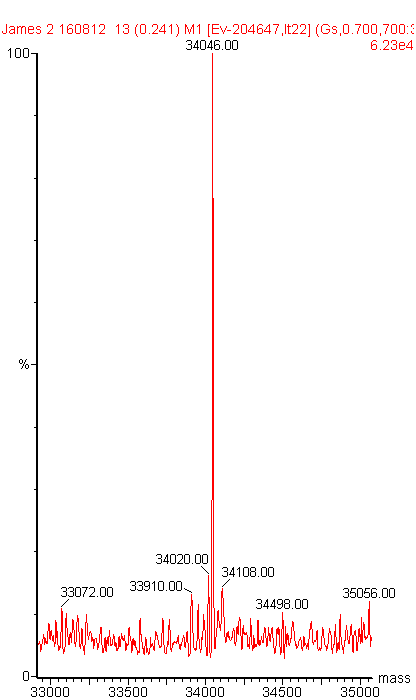


**Figure S8: MS (MALDI) to show single alkylation of mNat2 after treatment with 2-iodoacetamide.**

**(upper)** MS-MALDI of unmodified mNat2 (1 mg/mL in 25 µL aq Tris.HCl, pH 8 containing 5% DMSO (v/v)). **(lower)** MS-MALDI of modified mNat2 (1 mg/mL in 25 µL aq Tris.HCl, pH 8 containing 5% DMSO (v/v)).

**
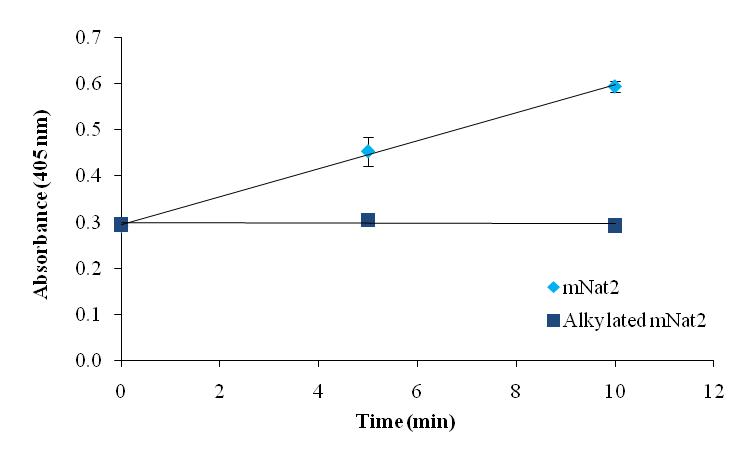

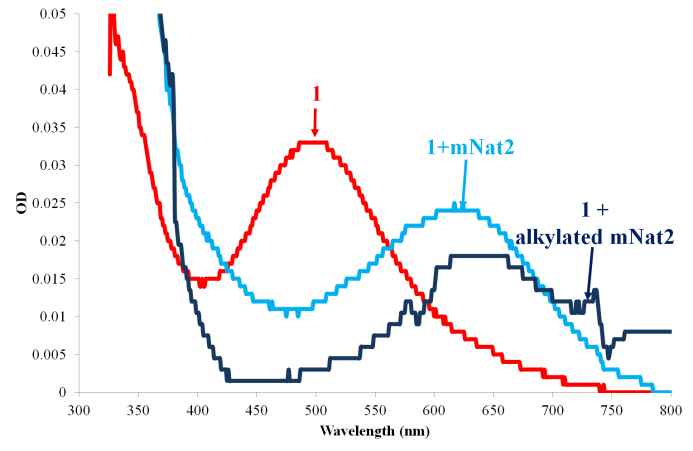
**

**Figure S9: Inactivation of mNat2 catalytic activity and optical properties of compound 1 with Cys68-alkylated mNat2.**

**(left)** AcCoA-hydrolysis assays with treated and untreated mNat2 (100 ng and 50 ng respectively) were carried out with AcCoA (400 μM) and pABA (500 μM) as arylamine substrate: ♦, untreated enzyme; ■, 2-iodoacetamide-treated enzyme. **(right)** Visible spectra of **1** (15 μM) in buffer (20 mM Tris.HCl, pH 8, 5% DMSO (v/v)) (red line), **1** (15 μM) with pure recombinant mNat2 (30 μM) in buffer (as above) (light blue line), **1** (15 μM) with Cys68-alkylated mNat2 (30 μM) in buffer (as above) (dark blue line).


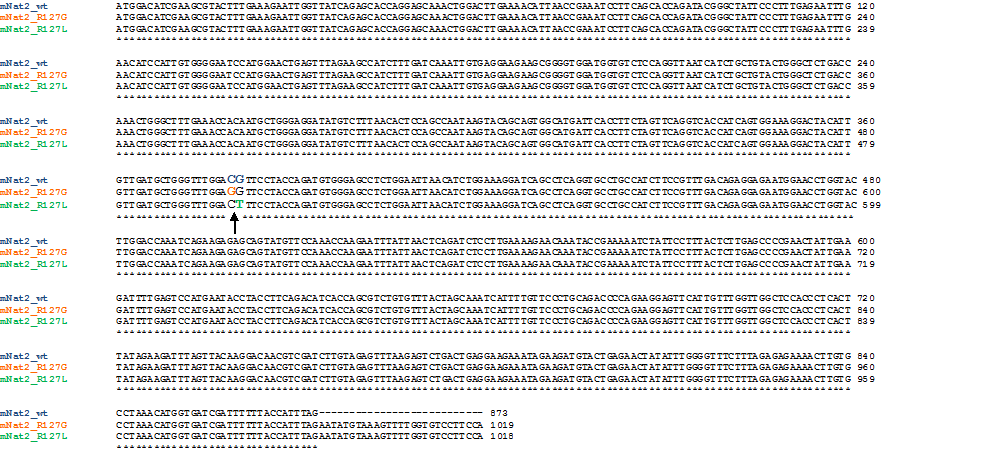


Figure S10: Multiple sequence alignment of wild-type *mNat2* gene and mutated *mNat2* genes.

The alignment was conducted by ClustalW [2]. Mutated nucleotides are highlighted in colour. The single site-directed mutagenesis were designed in order to obtain mNat2 mutants at residue R127. The mutated sequences encode R127G (orange) and R127L (green) mutants. * indicates nucleotide identity among all three genetic sequences. No additional mutations were generated during the site direct mutagenesis.


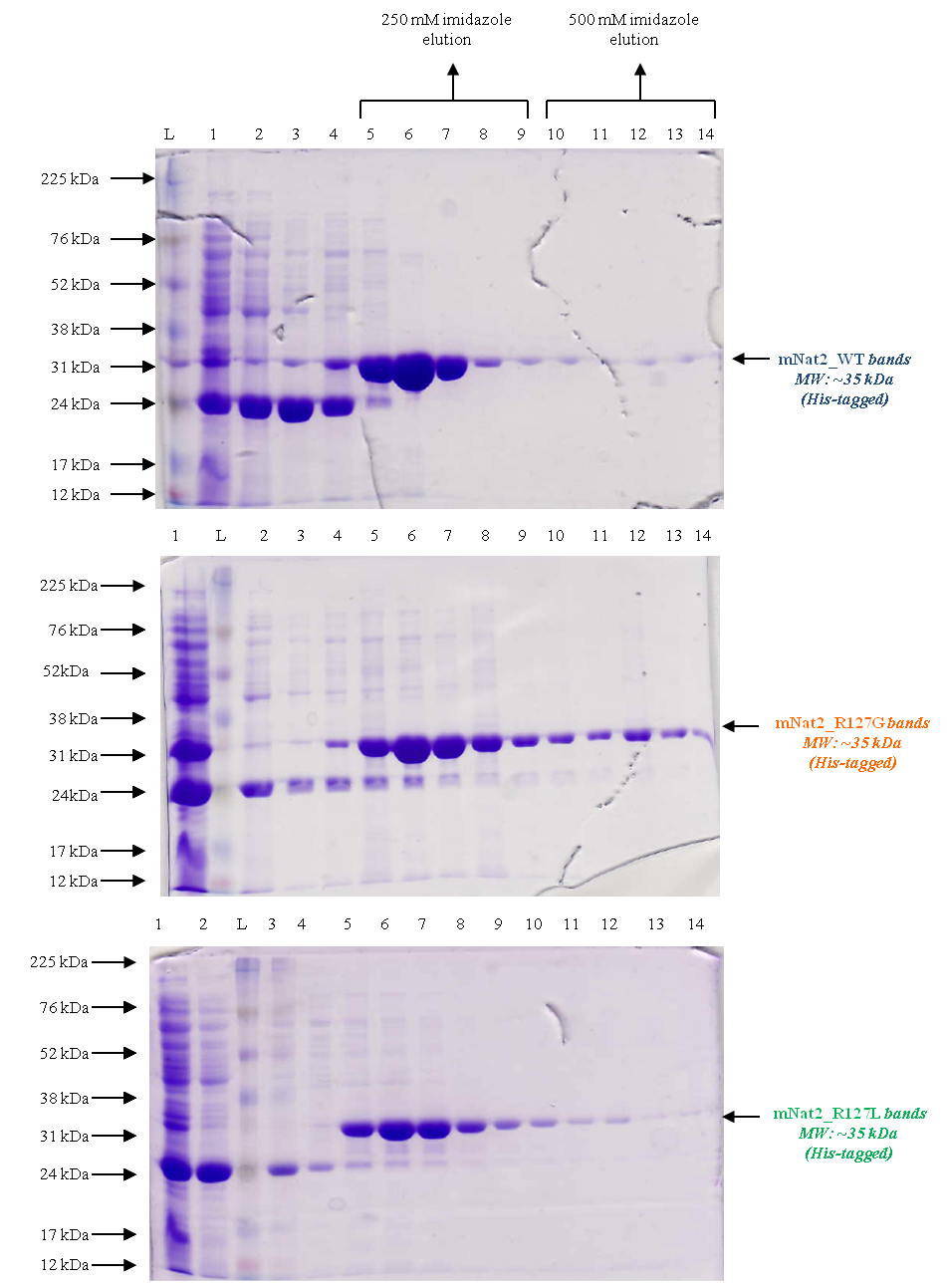


Figure S11: SDS-PAGE gels after purification of mNat2 variants *via* Ni-NTA affinity chromatography. Sodium dodecylsulfate polyacrylamide gel electrophoresis (SDS-PAGE) was used to separate and visualise protein samples as previously described [4] and performed by the method of Laemmli [5]. Coomassie Blue-stained 12% SDS-PAGE of each fraction from the Ni-NTA affinity purification process of recombinant mNat2_WT (upper), mNat2_R127G (middle), and mNat2_R127L (lower) are shown. The soluble fraction obtained after sonication of grown Rosetta(DE3)pLysS *E.coli* cells was loaded onto the Ni-NTA column and washed with buffered Tris.HCl solutions containing increasing concentrations of imidazole. Each well was loaded with 5 μL sample in 15 μL reduced gel loading buffer, except L (ladder) where 6 μL high range molecular weight Rainbow markers (GE Healthcare) were loaded. Lanes: 1 Unbound fraction, 2 0mM imidazole wash, 3 10mM imidazole wash, 4 25mM imidazole wash, 5-9 250mM imidazole washes, 10-14 500mM imidazole washes.


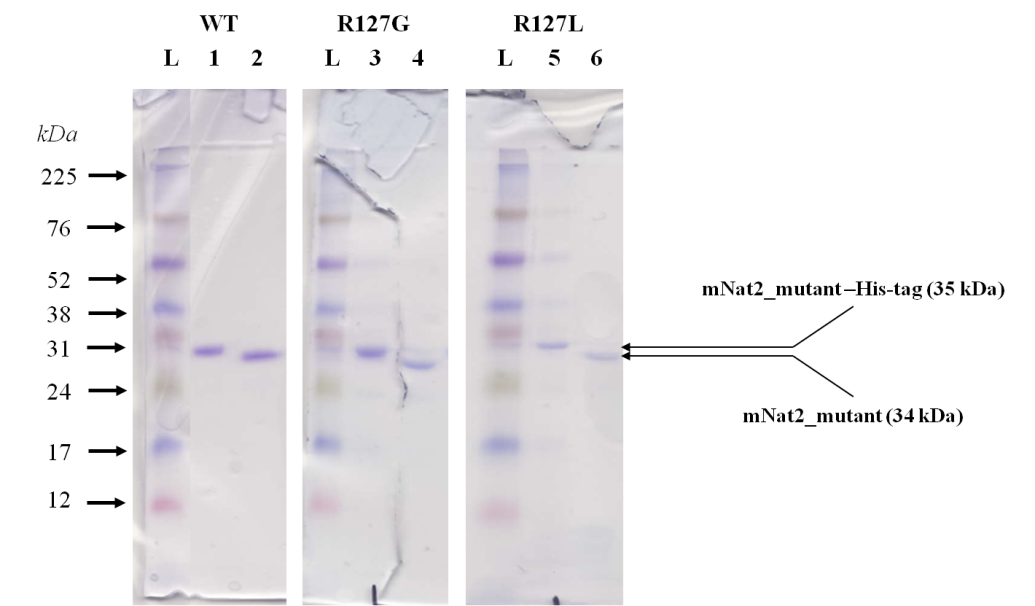


Figure S12: Thrombin cleavage of His-tag from recombinant mNat2 variants.

Coomassie Blue-stained 12% SDS-PAGE of fractions prior to and after the thrombin cleavage process of recombinant mNat2_WT, mNat2_R127G, and mNat2_R127L. Purified and concentrated His-tagged mNat2 variants were incubated with thrombin (5 U/mg protein) at 4 ºC for 16 h for complete His-tag cleavage. Each well was loaded with 5 μL sample in 15 μL reduced gel loading buffer. Lanes: L: ladder (6μl high range molecular weight Rainbow markers - GE Healthcare), 1, 3, 5: His-tagged mNat2 mutants; 2, 4, 6: Thrombin-treated mNat2 mutants.

**General Experimental**

**Chemicals** were purchased from Sigma-Aldrich UK, TCI UK, Apollo Scientific UK, Alfa Aesar UK, Fluorochem UK or Fisher Scientific UK unless otherwise stated. Where appropriate and if not stated otherwise, all reactions involving moisture-sensitive reagents were carried out under a nitrogen or argon atmosphere using standard vacuum line techniques and glassware that was flame-dried before use. Anhydrous DMF and anhydrous MeOH were purchased from Sigma-Aldrich UK in SureSeal^TM^ bottles and used without further purification. Water was purified by an Elix^®^ UV-10 system. All other solvents and reagents were used as supplied (analytical or HPLC grade) without prior purification.

**Organic layers** were dried over anhydrous MgSO_4_. Brine refers to a saturated aqueous solution of sodium chloride. *In vacuo* refers to the use of a rotary evaporator attached to a diaphragm pump. Pet ether refers to the fraction of petroleum spirit boiling between 30 and 40 °C, unless otherwise stated.

**Thin layer chromatography** was performed on Merck silica or alumina gel 60 F_254_ aluminium-supported thin layer chromatography sheets. Plates were visualised using UV light (254 nm), or thermal development after dipping in 1% aq. KMnO_4_ or ninhydrin.

**Flash column chromatography** was performed on Kieselgel 60 silica or alumina in a glass column, or on a Biotage SP4 flash column chromatography platform.

**Melting points** were recorded on a Gallenkamp Hot Stage apparatus and are uncorrected. Where relevant, the recrystallisation solvent is reported in parentheses.

**Infrared spectra** were recorded on a Bruker Tensor 27 FT-IR spectrometer, neat or as KBr discs (as stated). Selected characteristic peaks are reported in wavenumbers (cm^-1^).

**NMR spectra** were recorded on Bruker Avance spectrometers (DPX400, DQX400, AVII 500 or DRX500) in the deuterated solvent stated. The field was locked by external referencing to the relevant deuteron resonance. Chemical shifts (δ) are reported in parts per million (ppm) relative to tetramethylsilane (TMS) where δ_H_ (TMS) = 0.00 and δ_C_ (TMS) = 0.00. The multiplicity of each signal is indicated by: s (singlet); br. s (broad singlet); d (doublet); t (triplet); dd (doublet of doublets); td (triplet of doublets); tt (triplet of triplets); or m (multiplet). The prefix app. denotes that these coupling patterns are apparent. The number of protons or carbons (n) for a given resonance signal is indicated by nH or nC respectively. Coupling constants (*J*) are quoted in Hz and are reported to the nearest 0.1 Hz. The coupling constants are determined by analysis using ACD Labs software.

**Low-resolution mass spectra** were recorded on either a VG MassLab 20-250 or a Micromass Platform 1 spectrometer, operating in positive or negative mode, from solutions of methanol. Accurate mass measurements were run on either a Bruker MicroTOF internally calibrated with polyalanine, or a Micromass GCT instrument fitted with a Scientific Glass Instruments BPX5 column (15 m × 0.25 mm) using amyl acetate as a lock mass, by the mass spectrometry department of the Chemistry Research Laboratory, University of Oxford, UK. *m/z* values are reported in Daltons and followed by their percentage abundance in parentheses.

**Reverse-phase high-performance liquid chromatography** (RP-HPLC) was performed on a Gilson instrument equipped with Gilson 306 pumps, a Gilson 811C dynamic mixer, a Gilson 806 manometric module with automated sample injection on a Gilson 215 Liquid Handler, configured with a Gilson 819 valve actuator. 2 mg of the solid compound to be tested was dissolved in 1 mL of MeCN and 40 μL of this solution was automatically injected. Separations were performed on a Varian Omnisphere 5 C18 (analytical) column (5 μm particle size, 150.0 mm x 4.6 mm). All experiments were performed under gradient elution (eluent H_2_O containing 0.1% (v/v) TFA:MeCN 95:5 to 5:95 over 15 min. then isocratic for 10 min., except for compound **4**, when no TFA was added to the eluent). The flow rate was 1.0 mL/min. Detection was at a wavelength of 254 nm using a Gilson 170 Diode Array Detector. HPLC traces are displayed for the final compounds **3**, **4** and **5**.

**NAT activity** was determined by AcCoA hydrolysis assays. The NAT isoform (100 ng) was pre-incubated with *para*-amino benzoic acid (500 μM) in the assay buffer (5% DMSO/95% 20mM aq Tris.HCl, pH 8.0) for 5 min. at 37 °C in a 96-well plate (Corning). Pre-warmed AcCoA (400 μM, 37 °C) in assay buffer was added to start the reaction (final volume of 100 μL), which was allowed to proceed at 37 °C. Simultaneous quenching and color development was achieved by addition of 25 μL DTNB solution (5 mM DTNB in 100 mM aq Tris.HCl, 6.4 M guanidine.HCl, pH 7.5). The absorbance was immediately read at 405 nm using a plate reader (Tecan Sunrise). The rate of reaction was determined by reference to a standard curve and controls with buffer alone were carried out. For detection of inhibition, the inhibitors tested were dissolved in DMSO and the final percentage of DMSO in the assay was 5% (v/v). A range of 10 different concentrations of the inhibitor was selected from each inhibitor and IC_50_ values were estimated graphically from plots of specific activities *versus* inhibitor concentration by using Kyplot^®^ software. A dose-response function was used as a model of regression for the data and the curves were estimated by the method of the least squares analysis.

**Synthesis Schemes for the Preparation of Reported Compounds**

**Reagents and Conditions:** (i) 3 M aq NaOH, ^15^NH_4_Cl, 90 °C, 0.5 h; (ii) 2,3-dichloro-1,4-naphthoquinone **8** (0.77 eq.), Cs_2_CO_3_ (1 eq.), toluene, 180 °C, µw, 0.66 h; (ii) CeCl_3_.7H_2_O (1 eq.), toluene, RT, 90 min. then 3,5-dimethylaniline (3 eq.), 110 °C, 16 h.

**Reagents and Conditions:** (i) PhSO_2_NH_2_ (1 eq.), Cs_2_CO_3_ (1.4 eq.), DMF, RT, 5 h; (ii) CeCl_3_.7H_2_O (1 eq.), MeOH, RT, 90 min. then 3,5-dimethylaniline (3 eq.), 110 °C, 16 h; (iii) TMS-CHN_2­_ (1 eq.), MeOH (17 eq.), CH_2_Cl_2_, RT, 20 h.

**Reagents and Conditions:** (i) BnOH (2.2 eq.), Cs_2_CO_3_ (2.2 eq.), THF, reflux, 18 h; (ii) CeCl_3_.7H_2_O (1 eq.), MeOH, RT, 90 min. then 3,5-dimethylaniline (3 eq.), 110 °C, 16 h; (iii) H_2_, Pd/C 10% (0.2 eq.), MeOH, RT, 16 h; (iv) PhSO_2_Cl (excess), Et_3_N (3 eq.), RT, 16 h.

**Characterisation Data for Reported Compounds**

***N*-(3-(3’’,5’’-Dimethylphenylamino)-1,4-dioxo-1,4-dihydronaphthalen-2-yl)benzenesulfonamide 1{Laurieri, 2010 #5007}**

Naphthoquinone **10** (1.30 g, 3.75 mmol) was stirred with cerium trichloride heptahydrate (1.39 g, 3.75 mmol) in MeOH (10 mL) at RT for 1.5 h in a sealed microwave vial. 3,5-dimethylaniline (1.40 mL, 11.25 mmol) was added and the reaction mixture heated to 110 °C for 16 h. The solution was cooled to RT, NH_4_Cl (sat. aq., 50 mL) was added and the organic product was extracted with EtOAc (3 x
50 mL). The organic layer was collected, washed with brine (50 mL), dried, filtered and concentrated *in vacuo* to give the crude product. Purification *via* column chromatography on silica gel (eluent petroleum ether:acetone 90:10 to 50:50) and subsequent recrystallisation from boiling toluene gave 3-anilinosulfonamide **1** as a dark red solid (986 mg, 61%), which was subsequently recrystallised from boiling toluene (792 mg, 49%). mp 188-192 °C (toluene) (lit. [6] 178-182 °C (toluene)); δ_H_ (400 MHz, DMSO-*d*_6_) 2.22 (6H, s, 2 x Ar-*Me*), 6.59 (2H, s, H_2’’_ and H_6’’_), 6.67 (1H, s, H_4’’_), 7.34-7.40 (2H, m, H_3’_ and H_5’_), 7.47-7.51 (1H, m, H_4’_), 7.51-7.56 (2H, m, H_2’_ and H_6’_), 7.74-7.83 (3H, m, H_6_, H_7_ and H_5_ or H_8_), 8.00-8.04 (1H, m, H_5_ or H_8_), 8.79 (1H, s, N*H*), 9.06 (1H, s, N*H*); *m/z* (ESI^-^) 431 ([M-H]^-^, 100%).

***N*-(3-(3’’,5’’-Dimethylphenylamino)-1,4-dioxo-1,4-dihydronaphthalen-2-yl)^15^*N*-benzenesulfonamide 2**

^15^*N*-labelled naphthoquinone **9** (200 mg, 0.57 mmol) was stirred with cerium trichloride heptahydrate (87 mg, 0.23 mmol) in toluene (5 mL) at RT for 1.5 h in a sealed microwave vial. 3,5-Dimethylaniline (214 μL, 1.71 mmol) was added and the reaction mixture heated to 110 °C for 16 h. The solution was cooled to RT, sat. aq. NH_4_Cl (20 mL) was added and the organic product was extracted with EtOAc (3 x 20 mL). The organic layer was collected, washed with brine (20 mL), dried, filtered and concentrated *in vacuo* to give the crude product. Partial purification *via* column chromatography (eluent petroleum ether:acetone 75:25) gave 3-anilinosulfonamide **2** as a purple solid (179 mg, 72%), which was subsequently recrystallised from toluene (77 mg, 31%) to give a compound with physical and spectroscopic data consistent with **1**; *m/z* (ESI^-^) 432 ([M-H]^-^, 100%); HRMS (ESI^+^) C_24_H_20_N^15^NNaO_4_S^+^, ([M+Na]^+^) requires 456.1007; found 456.1014.

***N*-(3-(3’’,5’’-Dimethylphenylamino)-1,4-dioxo-1,4-dihydronaphthalen-2-yl)-*N*-methylbenzenesulfonamide 3**

Anhydrous CH_2_Cl_2_ (5 mL) and anhydrous methanol (537 μL, 13.37 mmol) were added to a microwave vial containing naphthoquinone **1** (340 mg, 0.787 mmol) sealed under argon. This mixture was stirred until **1** had dissolved completely. Trimethylsilyldiazomethane solution (400 μL, 2 M in hexanes, 0.787 mmol) was then carefully added to the solution, which was stirred at RT for 20 h. After this time, the reaction was quenched by adding 20% AcOH (10 mL), and then partitioned between EtOAc (50 mL) and sat. aq NaHCO_3_ (50 mL). The organic layer was separated and the aqueous phase extracted twice more with EtOAc (2 x 50 mL). The combined organic extracts were washed with brine (50 mL), dried, filtered and concentrated *in vacuo* to give the crude product. Purification *via* column chromatography on silica gel (eluent pet ether:EtOAc 95:5 to 80:20) gave N*-*methylated species **3** as an orange solid (194 mg, 55%). mp 217-219 °C; υ_max_ (neat) 3310, 2921, 1674, 1639; δ_H_ (500 MHz, DMSO-*d*_6_) 2.28 (6H, s, 2 x Ar-*Me*), 2.83 (3H, s, N-*Me*), 6.85 (3H, app s, H_2’’_, H_4’’_ and H_6’’_), 7.42-7.47 (2H, m, H_3’_ and H_5’_), 7.51-7.54 (2H, m, H_2’_ and H_6’_), 7.56-7.61 (1H, tt, *J* 7.3, 1.3, H_4’_), 7.74-7.80 (2H, m, H_6_ and H_8_), 7.80-7.85 (1H, m, H_7_), 8.03-8.06 (1H, dd, *J* 7.6, 1.0, H_5_), 9.16 (1H, s, N-*H*); δ_C_ (500 MHz, DMSO-*d*_6_) 20.8, 36.3, 116.4, 122.9, 125.7, 126.1, 126.9, 127.2, 128.6, 130.0, 132.3, 132.4, 132.8, 135.1, 136.7, 138.9, 138.9, 145.8, 178.5, 182.6; *m*/*z* (ESI^+^) 469 ([M+Na]^+^, 100%); HRMS (ESI^+^) C_25_H_22_N_2_NaO_4_S^+^ ([M+Na]^+^) requires 469.1192, found 469.1178.

**Methyl-*N*-3-(3’’,5’’-dimethylphenylamino)-1,4-dioxo-1,4-dihydronaphthalen-2-ylbenzenesulfonimidate 4**

Following the same procedure as for synthesis and purification of **3** above, *O-*methylated species **4** was formed as a purple solid (123 mg, 35%). mp 71-74 °C; υ_max_ (neat) 3323, 2921, 1647; δ_H_ (500 MHz, CDCl_3_) 2.16 (6H, s, 2 x Ar-*Me*), 3.54 (3H, s, O-*Me*), 6.47 (1H, s, H_4’’_), 6.62 (2H, s, H_2’’_ and H_6’’_), 7.20 (1H, s, N-*H*), 7.26-7.31 (2H, m, H_3’_ and H_5’_), 7.43-7.50 (3H, m, H_2’_, H_4’_ and H_6’_), 7.63 (1H, app. td, *J* 7.3, 1.3, H_6_), 7.67 (1H, app. td, *J* 7.3, 1.3, H_7_), 8.05 (1H, dd, *J* 7.3, 1.3, H_5_), 8.11 (1H, dd, *J* 7.3, 1.3, H_­8_); δ_C_ (500 MHz, CDCl_3_) 21.1, 55.7, 120.0, 124.9, 126.0, 126.3, 126.8, 127.0, 128.4, 130.7, 132.0, 132.1, 132.7, 133.9, 134.7, 137.7, 138.8, 139.3, 179.1, 182.7; the identity of **4** was supported by nOe analysis - irradiation of O-*Me* shows a strong signal enhancement of H_2’_/H_6’_ and H_2’’_/H_6’’_; *m*/*z* (ESI^+^) 469 ([M+Na]^+^, 100%); HRMS (ESI^+^) C_25_H_22_N_2_NaO_4_S^+^ ([M+Na]^+^) requires 469.1192, found 469.1184.

**3-((3’’,5’’-Dimethylphenyl)amino)-1,4-dioxo-1,4-dihydronaphthalen-2-ylbenzenesulfonate 5**

Naphthoquinone **13** (40 mg, 0.137 mmol) was added to benzenesulfonyl chloride (5 mL) and triethylamine (57 µL, 0.410 mmol) and stirred at RT for 16 h. The reaction mixture was quenched with MeOH (8 mL) and then partitioned between HCl (1 M aq., 20 mL) and EtOAc (20 mL). The organic layer was separated and the aqueous phase extracted twice more with EtOAc (2 x 20 mL). The combined organic extracts were washed with brine (20 mL), filtered and concentrated *in vacuo* to give the crude product. Purification *via* column chromatography on silica gel (eluent petroleum ether:EtOAc 95:5 to 75:25) gave sulfonate ester **5** as a red solid (51 mg, 86%), which was subsequently recrystallised from boiling methanol (30 mg, 51%). mp 200-203 °C (MeOH); υ_max_ (neat) 3288, 1680, 1616, 1598, 1576, 1530; δ_H_ (500 MHz, CDCl_3_) 2.22 (6H, s, 2 x Ar-*Me*), 6.51 (2H, s, H_2’’_ and H_6’’_), 6.70 (1H, s, H_4’’_), 7.35 (2H, t, *J* 7.9, H_3’_ and H_5’_), 7.48 (1H, s, -N*H*), 7.54-7.62 (3H, m, J_2’_, H_4’_ and H_6’_), 7.70 (1H, t, *J* 7.4, H_7_), 7.79 (1H, t, *J* 7.4, H_6_), 8.13 (1H, d, *J* 7.4, H_8_), 8.18 (1H, d, *J* 7.4, H_5_); δ_C_ (125 MHz, CDCl_3_) 21.2, 120.6, 126.8, 126.8, 126.9, 127.2, 127.9, 128.6, 129.8, 131.8, 132.9, 133.5, 135.4, 135.8, 136.3, 136.3, 137.9, 177.2, 182.2; HMBC analysis confirms that the product is the 1,4-dione and not the 1,2-dione as the carbonyl resonance at 182.2 ppm shows interactions with both –N*H* and C_5_-*H*; *m/z* (ESI^+^) 456 ([M+Na]^+^, 100%); HRMS (ESI^+^) C_24_H_19_NNaO_5_S ([M+Na]^+^) requires 456.0876, found 456.0859.

**^15^*N*-benzenesulfonamide 7**

Benzenesulphonyl chloride **6** (256 μL, 2.01 mmol) was added dropwise to a NaOH solution (3 M aq., 10 mL) containing ^15^*N*-labelled ammonium chloride and the resulting mixture was heated at 90 °C for 30 min. before being cooled to RT in a procedure adapted from [7]. Filtration of the reaction mixture gave sulfonamide **7** as a white crystalline solid (231 mg, 73%). mp 152-154 °C; δ_H_ (400 MHz, Methanol-*d*_4_) 7.51-7.66 (3H, m), 7.87-7.96 (2H, m), N*H_2_* not observed; *m/z* (ESI^-^) 157 ([M-H]^-^, 100%).

***N*-(3-Chloro-1,4-dioxo-1,4-dihydronaphthalen-2-yl)^15^*N*-benzenesulfonamide 9**

2,3-Dichloronaphthalene-1,4-dione **8** (221 mg, 0.97 mmol), ^15^*N*-labelled benzenesulfonamide **7** (200 mg, 1.26 mmol) and Cs_2_CO_3_ (411 mg, 1.26 mmol) were subjected to microwave irradiation in a sealed vessel at 170 °C in toluene (5 mL) over 40 min. The reaction mixture was partitioned between CH_2_Cl_2_ (40 mL) and HCl (1 M aq., 40 mL) and the organic extract was washed with brine (40 mL), dried, and concentrated *in vacuo* to give a crude ^15^*N*-labelled sample of sulfonamide **9**; *m/z* (ESI^-^) 347 ([M-H]^-^, 100%); other spectroscopic data as reported for **10**.

***N*-(3-Chloro-1,4-dioxo-1,4-dihydronaphthalen-2-yl)benzenesulfonamide 10**

2,3-Dichloronaphthalene-1,4-dione **8** (1.00 g, 4.4 mmol), benzenesulfonamide (0.69 g, 4.4 mmol) and Cs_2_CO_3_ (2.00 g, 6.16 mmol) were stirred in DMF (10 mL) in a sealed microwave vial at RT for 16 h. HCl (1 M, 100 mL) was added and the organic product extracted with EtOAc (100 mL). The organic layer was separated and the aqueous phase extracted twice more with EtOAc (2 x 100 mL). The combined organic extracts were washed with brine (100 mL), dried, filtered and concentrated *in vacuo* to give the crude product. Purification *via* column chromatography on silica gel (eluent pet ether:acetone 75:25) gave sulfonamide **10** as a yellow solid (1.392 g, 91%). mp 219-223 °C (lit. [6] > 250 °C); δ_H_ (400 MHz, DMSO-*d*_6_) 7.56-7.64 (2H, m, H_3’_ and H_5’_), 7.66 (1H, app. t, *J* 7.1, H_4’_), 7.81-7.90 (2H, m, H_6_ and H_7_), 7.91-7.98 (3H, H_2’_, H_6’_ and H_5_ or H_8_), 8.01-8.06 (1H, m, H_5_ or H_8_); *m/z* (ESI^-^) 346 ([M(^35^Cl)-H]^-^, 100%).

**2,3-Bis(benzyloxy)naphthalene-1,4-dione 11**

2,3-Dichloronaphthalene-1,4-dione **8** (1.00 g, 4.40 mmol), benzyl alcohol (1.00 mL, 9.69 mmol) and Cs_2_CO_3_ (3.16 g, 9.69 mmol) were stirred in THF (20 mL) and heated to reflux for 18 h. HCl (1 M, 100 mL) was then added and the organic product extracted with EtOAc (3 x 100 mL). The combined organic extracts were washed with brine (100 mL), dried, filtered and concentrated *in vacuo* to give the crude reaction mixture. Purification *via* column chromatography on alumina gel (eluent petroleum ether:EtOAc 99:1 to 95:5) gave 2,3-bisbenzyloxynaphthoquinone **11** as a yellow solid (503 mg, 39%). This was used without further purification in the next step. mp 87-91 °C; υ_max_ (neat) 3033, 1660, 1595, 1573; δ_H_ (500 MHz, DMSO-*d*_6_) 5.26 (4H, s, 2 x –C*H*_2_), 7.29-7.52 (10H, m, H_2’_, H_3’_, H_4’_, H_5’_, H_6’_, H_2’’_, H_3’’_, H_4’’_, H_5’’_ and H_6’’_), 7.79-8.00 (2H, m, (H_6_, H_7_, H_5_ and H_8_); δ_C_ (125 MHz, DMSO-*d*_6_) 75.6, 126.6, 129.0, 129.1, 129.3, 131.4, 135.0, 137.6, 148.5, 182.4; *m/z* (ESI^+^) 393 ([M+Na]^+^, 100%); HRMS (ESI^+^) C_24_H_18_NaO_4_^+^ ([M+Na]^+^) requires 393.1097, found 393.1082.

**2-(Benzyloxy)-3-((3’’,5’’-dimethylphenyl)amino)naphthalene-1,4-dione 12**

Naphthoquinone **11** (250 mg, 0.676 mmol) was stirred with cerium trichloride heptahydrate (252 mg, 0.676 mmol) in MeOH (8 mL) at RT for 1.5 h in a sealed microwave vial. 3,5-dimethylaniline (252 µL, 2.028 mmol) was added and the reaction mixture heated to 110 °C for 16 h. The solution was cooled to RT, NH_4_Cl (sat. aq., 50 mL) was added and the organic product was extracted with EtOAc (3 x 50 mL). The combined organic extracts were washed with brine (50 mL), dried, filtered and concentrated *in vacuo* to give the crude product. Purification *via* column chromatography on silica gel (eluent pet ether:EtOAc 95:5 to 75:25) gave 3-anilinonaphthoquinone **12** as a red solid (227 mg, 88%), which was subsequently recrystallised from boiling methanol (110 mg, 43%). mp 152-153 °C (MeOH); υ_max_ (neat) 3312, 1705, 1663, 1637, 1597, 1570, 1521; δ_H_ (500 MHz, DMSO-*d*_6_) 2.18 (6H, s, 2 x Ar-*Me*), 4.75 (2H, s, -C*H*_2_Ph), 6.67 (1H, s, H_4’’_), 6.72 (2H, s, H_2’’_ and H_6’’_), 6.89-6.92 (2H, m, H_2’_ and H_6’_), 7.16-7.24 (3H, m, H_3’_, H_4’_ and H_5’_), 7.77 (1H, td, *J* 7.8, 1.2, H_6_ or H_7_), 7.85 (1H, td, *J* 7.8, 1.2, H_6_ or H_7_), 7.97-8.01 (2H, m, H_5_ and H_8_), 8.42 (1H, s, -N*H*); δ_C_ (125 MHz, DMSO-*d*_6_) 20.9, 72.7, 120.2, 124.7, 125.5, 125.8, 127.5, 127.8, 130.1, 131.7, 132.9, 134.6, 135.6, 136.8, 136.8, 138.3, 139.8, 178.7, 182.7; *m/z* (ESI^+^) 252 (100%), 384 ([M+H]^+^, 40%), 406 ([M+Na]^+^, 50%); HRMS (ESI^+^) C_25_H_21_NNaO_3_^+^ ([M+Na]^+^) requires 406.1414. found 406.1395.

**3-((3’’,5’’-Dimethylphenyl)amino)-2-hydroxynaphthalene-1,4-dione 13**

Naphthoquinone **12** (200 mg, 0.522 mmol) and 10% Pd/C (11 mg, 0.104 mmol) were stirred in MeOH (15 mL) in a sealed microwave vial under H_2_ for 16 h. The mixture was filtered through Celite^®^ and the filtrate concentrated *in vacuo* to give the crude product. Purification *via* column chromatography on silica gel (eluent petroleum ether:acetone 90:10 to 75:25) gave 2-hydroxynaphthoquinone **13** as a purple solid (49 mg, 32%). mp 212-215 °C; υ_max_ (neat) 3316, 1655, 1640, 1594, 1572, 1517; δ_H_ (500 MHz, CDCl_3_) 2.19 (6H, s, 2 x Ar-*Me*), 6.50 (3H, app s, H_2’_, H_4’_ and H_6’_), 7.74-7.81 (3H, m, -N*H*, H_6_ and H_7_), 7.94-7.98 (2H, m, H_5_ and H_8_), 10.09 (1H, br. s, -O*H*); δ_C_ (125 MHz, CDCl_3_) 21.1, 117.4, 122.3, 125.3, 125.7, 125.9, 130.6, 130.8, 133.5, 133.7, 136.6, 141.1, 141.6, 179.2, 182.1; *m/z* (ESI^-^) 292 ([M-H]^-^, 100%); HRMS (ESI^-^) C_16_H_14_NO_3_^-^ ([M-H]^-^) requires 292.0979, found 292.0976.

**NMR Spectra and HPLC Traces for Reported Compounds**

**Compound 1 (^1^H-NMR)**

**Compound 2 (^1^H-NMR and ^13^C-NMR)**

**Compound 3 (^1^H-NMR, ^13^C-NMR and HPLC Trace)**

**Compound 4 (^1^H-NMR, ^13^C-NMR, nOe Experiments and HPLC Trace)**

**Compound 5 (^1^H-NMR, ^13^C-NMR, HMBC and HPLC Trace)**

**Compound 7 (^1^H-NMR)**

**Compound 10 (^1^H-NMR)**

**Compound 11 (^1^H-NMR and ^13^C-NMR)**

**Compound 12 (^1^H-NMR and ^13^C-NMR)**

**Compound 13 (^1^H-NMR and ^13^C-NMR)**

**Supplementary Table**

**mNat2_WT**

| **Fraction** | **Volume (ml)** | **Total Protein***^a^* **(mg)** | **Total Activity***^b^* **(μM.min^-1^)** | **Specific Activity (μM.min^-1^.mg^-1^)** | **Purification Fold** | **Yield (%)** |
| --- | --- | --- | --- | --- | --- | --- |
| Soluble Cell Lysate | 20 | 1097 | 5936 | 5.41 | 1.00 | 100 |
| Unbound Lysate | 20 | 653 | 374 | 0.57 | 0.11 | 6 |
| 0mM imidazole wash | 10 | 163 | 18 | 0.11 | 0.02 | 0 |
| 10mM imidazole wash | 10 | 85 | 58 | 0.68 | 0.13 | 1 |
| 25mM imidazole wash | 10 | 120 | 480 | 4.00 | 0.74 | 8 |
| 250mM imidazole wash | 18.4 | 51 | 4096 | 79.78 | 14.75 | **69** |
| 500mM imidazole wash | 20.5 | 3 | 39 | 14.50 | 2.68 | 1 |

**mNat2_R127G**

| **Fraction** | **Volume (ml)** | **Total Protein***^a^* **(mg)** | **Total Activity***^b^* **(μM.min^-1^)** | **Specific Activity (μM.min^-1^.mg^-1^)** | **Purification Fold** | **Yield (%)** |
| --- | --- | --- | --- | --- | --- | --- |
| Soluble Cell Lysate | 18.5 | 934 | 1465 | 1.57 | 1.00 | 100 |
| Unbound Lysate | 18 | 632 | 433 | 0.69 | 0.44 | 30 |
| 0mM imidazole wash | 10 | 104 | 15 | 0.15 | 0.09 | 1 |
| 10mM imidazole wash | 10.25 | 22 | 25 | 1.17 | 0.75 | 2 |
| 25mM imidazole wash | 10.25 | 21 | 43 | 2.04 | 1.30 | 3 |
| 250mM imidazole wash | 20.25 | 95 | 438 | 4.60 | 2.93 | **30** |
| 500mM imidazole wash | 20.25 | 28 | 197 | 7.07 | 4.51 | 13 |

**mNat2_R127L**

| **Fraction** | **Volume (ml)** | **Total Protein***^a^* **(mg)** | **Total Activity***^b^* **(μM.min^-1^)** | **Specific Activity (μM.min^-1^.mg^-1^)** | **Purification Fold** | **Yield (%)** |
| --- | --- | --- | --- | --- | --- | --- |
| Soluble Cell Lysate | 18 | 1282 | 44 | 0.03 | 1.00 | 100 |
| Unbound Lysate | 17.5 | 865 | 6 | 0.01 | 0.19 | 13 |
| 0mM imidazole wash | 11 | 198 | 1 | 0.00 | 0.10 | 2 |
| 10mM imidazole wash | 10 | 60 | 0 | 0.00 | 0.00 | 0 |
| 25mM imidazole wash | 10.25 | 11 | 0 | 0.00 | 0.00 | 0 |
| 250mM imidazole wash | 19.25 | 48 | 29 | 0.61 | 18.05 | **68** |
| 500mM imidazole wash | 19 | 2 | 3 | 2.10 | 61.74 | 8 |

Table S1: Purification of mNat2 variants using Ni-NTA affinity chromatography.

The soluble fractions obtained following cell sonication were loaded onto the Ni-NTA affinity column and washed with increasing concentrations of imidazole, and volumes of column eluates were measured. Yield and purification fold of mNat2_WT, mNat2_R127G and mNat2_R127L were calculated. ^a^ - Protein concentrations were measured using the Bradford assay in triplicate (given to the nearest mg).^b^ - Activity was measured using the AcCoA-hydrolysis assay in triplicate samples as outlined in Materials and Methods (given to the nearest μM/min). The linearity of the activity over time was checked.

**References**

[1] Wu H, Dombrovsky L, Tempel W, Martin F, Loppnau P, et al. (2007) Structural basis of substrate-binding specificity of human arylamine N-acetyltransferases. J Biol Chem 282: 30189-30197.

[2] Thompson JD, Higgins DG, Gibson TJ (1994) CLUSTAL W: improving the sensitivity of progressive multiple sequence alignment through sequence weighting, position-specific gap penalties and weight matrix choice. Nucleic Acids Res 22: 4673-4680.

[3] Gouet P, Courcelle E, Stuart DI, Métoz F. (1999) ESPript: multiple sequence alignments in PostScript. Bioinformatics 15: 305-308.

[4] Sambrook J, Fritsch EF, Maniatis T (1989) Molecular Cloning. A Laboratory Manual. Cold Spring Harbor, NY: Cold Spring Harbor Laboratory Press.

[5] Laemmli UK (1970) Cleavage of structural proteins during the assembly of the head of bacteriophage T4. Nature 227: 680-685.

[6] Laurieri N, Crawford MH, Kawamura A, Westwood IM, Robinson J, et al. (2010) Small molecule colorimetric probes for specific detection of human arylamine N-acetyltransferase 1, a potential breast cancer biomarker. J Am Chem Soc 132: 3238-3239.

[7] Salvatore BA, Prestegard, JH (1998) Synthesis of a 15N, 13C-labeled lactam analog of a GM4-lactone cell-surface glycolipid. Tetrahedron Lett 39: 9319-9322.
